# Single Channel Based Interference‐Free and Self‐Powered Human–Machine Interactive Interface Using Eigenfrequency‐Dominant Mechanism

**DOI:** 10.1002/advs.202302782

**Published:** 2024-01-29

**Authors:** Sen Ding, Dazhe Zhao, Yongyao Chen, Ziyi Dai, Qian Zhao, Yibo Gao, Junwen Zhong, Jianyi Luo, Bingpu Zhou

**Affiliations:** ^1^ Joint Key Laboratory of the Ministry of Education Institute of Applied Physics and Materials Engineering University of Macau Avenida da Universidade, Taipa Macau 999078 China; ^2^ Department of Electromechanical Engineering University of Macau Avenida da Universidade, Taipa Macau 999078 China; ^3^ Research Center of Flexible Sensing Materials and Devices School of Applied Physics and Materials Wuyi University Jiangmen 529020 China; ^4^ Shenzhen Shineway Technology Corporation Shenzhen Guangdong 518000 China

**Keywords:** damped oscillation, eigenfrequency, human–machine interaction, interference‐free, self‐powered

## Abstract

The recent development of wearable devices is revolutionizing the way of human–machine interaction (HMI). Nowadays, an interactive interface that carries more embedded information is desired to fulfill the increasing demand in era of Internet of Things. However, present approach normally relies on sensor arrays for memory expansion, which inevitably brings the concern of wiring complexity, signal differentiation, power consumption, and miniaturization. Herein, a one‐channel based self‐powered HMI interface, which uses the eigenfrequency of magnetized micropillar (MMP) as identification mechanism, is reported. When manually vibrated, the inherent recovery of the MMP causes a damped oscillation that generates current signals because of Faraday's Law of induction. The time‐to‐frequency conversion explores the MMP‐related eigenfrequency, which provides a specific solution to allocate diverse commands in an interference‐free behavior even with one electric channel. A cylindrical cantilever model is built to regulate the MMP eigenfrequencies via precisely designing the dimensional parameters and material properties. It is shown that using one device and two electrodes, high‐capacity HMI interface can be realized when the magnetic micropillars (MMPs) with different eigenfrequencies have been integrated. This study provides the reference value to design the future HMI system especially for situations that require a more intuitive and intelligent communication experience with high‐memory demand.

## Graphical abstract

A single channel based human–machine interactive interface is reported. The interface is composed of magnetized micropillars (MMPs) and a flexible coil. By precisely regulating eigenfrequency of various MMPs, different signals or commands can be transmitted with only 1 channel in form of induced current, which will significantly enlarge the capacity and improve the wearability of devices.

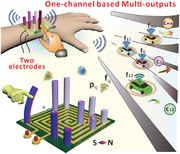

## 1 Introduction

The emergence of flexible and wearable electronics has recently aroused extensive interest from academic to industry society.^[^ ^1^, ^2^, ^3^, ^4^, ^5^, ^6^ ^]^ Apart from the healthcare monitoring,^[^ ^7^, ^8^, ^9^, ^10^, ^11^, ^12^ ^]^ wearable devices have also exhibited promising potentials as the platform for human–machine interaction (HMI).^[^ ^13^, ^14^, ^15^, ^16^, ^17^ ^]^ As a medium of communication between human beings and electric terminals, the HMI interface serves as an important channel to convert physiological signals into electrical signals, such as mechanical forces,^[^ ^18^ ^]^ body temperature,^[^ ^19^ ^]^ sweat,^[^ ^20^ ^]^ and sound,^[^ ^21^ ^]^ etc. Nowadays, wearable HMI systems have been demonstrated for applications in Internet of things (IoT), soft robotics, and virtual reality (VR), etc.^[^ ^22^, ^23^, ^24^, ^25^, ^26^ ^]^ With the continuous efforts, a sorts of mechanisms and approaches have also been successfully developed to realize the signal perception and conversion, including capacitive,^[^ ^27^ ^]^ resistive,^[^ ^28^ ^]^ triboelectric,^[^ ^29^ ^]^ optical,^[^ ^30^ ^]^ and magnetic,^[^ ^31^, ^32^, ^33^ ^]^ etc.

Specifically, the rapid development of IoT and artificial intelligence has now required a more effective HMI system to bridge the gaps between human and electric terminals.^[^ ^34^ ^]^ To broaden the communication memory, one straightforward approach is to integrate device arrays. However, unlike rigid electronic materials, high‐level integration is still a problem for flexible and wearable electronics.^[^ ^35^ ^]^ Furthermore, the number of hardware configurations and signal wires increases along with the number of components in a flexible HMI interface, and the array inevitably brings the concern of portability and system complexity. From this perspective, it is particularly important to improve the information capability of individual device.^[^ ^36^, ^37^, ^38^ ^]^ For example, Dai et al. proposed a flexible ternary sensor which can precisely perceive the bi‐directional stimuli with non‐overlapping response.^[^ ^39^ ^]^ Upon inward bending, the optimized microstructures enabled more contact points for resistance decrease, while an outward bending generated increased resistance due to the applied strain. Using photolithography and thermal deposition, An et al. explored the response of metallic gratings to IR radiation from human hand for non‐contact HMI, which can produce multiple commands based on the design of grating periods and duty cycles.^[^ ^40^ ^]^ Recently, triboelectric hybrid coder was demonstrated, which combines the single‐electrode and contact‐separation mode to identify touch and press for distinguishable coding.^[^ ^41^ ^]^ To date, however, to simply include a coding library into a single device for high‐capacity flexible and wearable HMI is still challenging.

**Figure 1:**
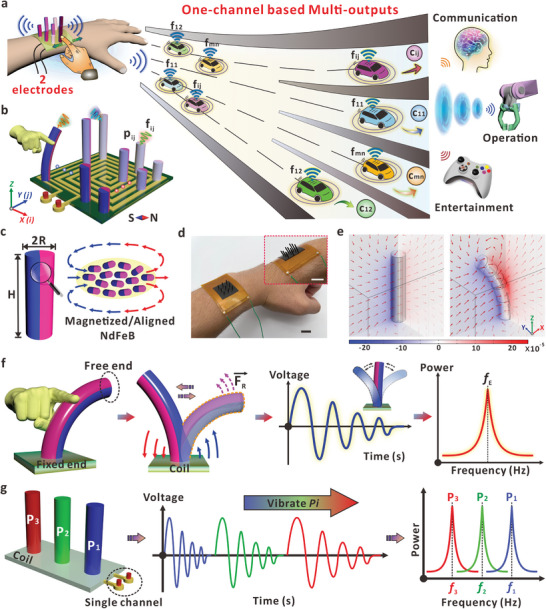
Design of flexible MMPs for eigenfrequency‐based HMI. a) Schematic diagram of human–machine interaction based on flexible and magnetized micropillars (MMPs). Signals with different eigenfrequencies are related with the MMP, which can be applied and encoded with commands for broad applications in HMI. Even though only one channel is used, the specific eigenfrequency (f_ij_) can be precisely allocated for target commands (c_ij_) without interference. b) Schematic diagram of the interface that contains magnetic micropillar array (MMPA) with one coil substrate. Eigenfrequency (f_ij_) of the MMP can be produced via vibrating the micropillars (p_ij_), which is encoded with related commands for HMI. The color of the micropillars indicates the elastic modulus (*E*) of the MMPA was tuned. c) Schematic diagram of a single magnetized micropillar. The height of the micropillar is *H* and the diameter of the cross‐sectional circle is *2R*. The right picture shows the schematic diagram of aligned NdFeB particles inside the pillar after magnetization. d) Optical images of the whole device with different MMPs on the human wrist. All scale bars in the images are 1 cm. e) The simulation result of surrounding magnetic field around single magnetized micropillar. The slices indicate the magnetic scalar potential (A), and the arrow volume is related with the magnetic flux density. f) Process to generate the eigenfrequency based on the damped oscillation of an individual MMP via finger‐induced deformation. The Prony analysis method was applied here to convert the electrical signals from time to frequency domain. g) Schematic diagram of the assembly with three specific MMPs of different eigenfrequencies. When the MMPs were vibrated consecutively, the voltage signals were captured via the single channel and converted to the distinguishable frequencies that can be identified by the terminal.

In nature, many existent elastic bodies, e.g. spring‐block system, and cantilever beam, can freely vibrate under a specific eigenfrequency that is mainly determined by the inherent properties.^[^ ^42^, ^43^ ^]^ Inspired by this phenomenon, we consider that the design and establishment of an eigenfrequency library can be potentially applied to encode identifiable commands/signals without interference for HMI. Here, we report a flexible and wearable HMI interface, which is dominant by the eigenfrequency of a specific magnetized micropillar (MMP) using the cylindrical cantilever model.^[^ ^44^, ^45^, ^46^ ^]^ When the MMP was deformed, the inherent recovery would cause the damped oscillation toward the equilibrium position. Based on Faraday's Law of Induction, the oscillation and corresponding variation of localized magnetic flux can be electrically perceived by the induced electromotive force (EMF)/electrical current in the conductive coil underneath.^[^ ^47^, ^48^ ^]^ Compared with wearable HMI interface using the capacitive or resistive sensors, the principle of electromagnetic induction allows the MMP to function in a self‐powered manner without providing extra power supply to the device.^[^ ^23^, ^28^ ^]^ More importantly, the conversion of electrical signals from time to frequency domain would figure out a specific eigenfrequency, which is mainly determined by the intrinsic property of the micropillar. On this basis, as depicted in **Figure** **1**a, the integration of MMPs with different eigenfrequencies on one device can enable a high‐capacity HMI system for applications from information communication, robotic operation, to daily entertainment. Unlike conventional addressing‐based multiple command system, one single channel is required here because the specific eigenfrequencies can provide the non‐overlapping signals to precisely allocate subsequent commands without interference. Through the theoretical model, simulation, and experimental validation, we prove that the eigenfrequencies can be controllable via tuning the parameters of micropillar height, material modulus, or density, etc. Moreover, the MMPs are robust, and the stable eigenfrequency production ensures the reliability of eigenfrequency‐dominant mechanism as an effective avenue to trigger intended command by one piece of device.

## 2 Results and Discussion

### 2.1 Design Principle

The design principle to apply MMP eigenfrequencies for effective HMI is provided in Figure 1a,b, which show that the micropillar arrays can be assembled together and only one electrical connection is required for signal collection. As discussed subsequently, the dimension and material property of the micropillars can both affect the eigenfrequency values. It is thus possible that the micropillars can be assembled with different heights, mechanical strength, or density, to realize the control of eigenfrequency. Via precisely designing the MMPs, the specific eigenfrequencies (f_ij_) can be accompanied with the oscillation of the micropillars (p_ij_), which can carry the embedded command (c_ij_) to complete the intended HMI process. Consequently, with the integrated MMP array containing *m* rows and *n* columns, a coil device can totally produce command capacity of *m* × *n*. Note that even the electrical signals are captured using the same channel, the specific eigenfrequency can be accurately identified to allocate related commands for communication. For example, when the micropillars P_12_ and P_11_ were vibrated, they will generate the eigenfrequency signals of f_12_ and f_11_, respectively. As the values of eigenfrequency are different, the mechanical inputs to vibrate specific MMPs can finally be transmitted and converted to the corresponding commands of c_12_ and c_11_ for HMI. The micropillars, composed of NdFeB particles, Polydimethylsiloxane (PDMS), and Ecoflex composite, were prepared based on a specific mass ratio. Detailed fabrication methodology of the MMPs and the conductive coil underneath are demonstrated in Figure S1a (Supporting Information) and Experimental Section. Optical images of the PMMA mold and the copper coil are discussed in Figure S1b,c, (Supporting Information) respectively. Figure S2 (Supporting Information) shows the Scanning Electron Microscopy (SEM) and Electron Dispersive Spectroscopy (EDS) result of the MMP and NdFeB particles, which indicates the uniform distribution of different elements within the matrix. In this work, the height and the radius of the micropillar were defined as *H* and *R*, respectively (Figure 1c). After the magnetization along the in‐plane direction, the embedded NdFeB particles aligned within the polymer base and the micropillar could serve as a flexible magnet with defined south (S) and north (N) poles. Due to high energy product of NdFeB,^[^ ^49^ ^]^ the MMP shows a large remanent magnetization with excellent flexibility as indicated by the magnetic hysteresis curvature of NdFeB/silicone polymer composite (Figure S3, Supporting Information). Thanks to the flexibility of the conductive copper coil and the MMP assembly, the device could be attached to the human skin for the wearable human–machine interactions. Figure 1d shows the customized device on the human wrist. As shown in the inset, both the MMPs and the coil can be properly bent according to the human wrist. The dimension of the coil is 3.3 cm × 4.8 cm, and the area of the sample integrated with nine MMPs is 1.9 cm × 1.9 cm. The flexibility of the micropillars not only results in excellent wearable performance, but more importantly, the MMP can serve as a flexible magnet to deform and vibrate for signal generation. The inset also shows that the micropillars on one device can be prepared with different heights, which was realized by defining the depth of the microholes in the mold (Figure S1b, Supporting Information). Figure 1e exhibits the simulation results of the magnetic field distribution around an MMP before and after the deformation (Experimental Section provides more details about the simulation setting). The magnetization direction is along the X‐axis of the micropillar, which is consistent with the experimental result. Thanks to the flexibility of the micropillar, the surrounding magnetic field could also alter during the deformation, thus providing the magnetic flux variation that can be perceived by the coil underneath. The schematic diagram in Figure 1f further demonstrates the signal generation and analysis process for the eigenfrequency‐based interactive interface. Owing to the flexibility of the MMP, the finger sweeping would impose the torque and cause the deformation of the micropillar at the free end. Once the mechanical constrain was released, the inherent restoring force (*F_R_*) of the micropillar causes the instant vibration of the MMP. With the oscillation around the equilibrium position, the magnetic field distribution above the coil changes simultaneously.^[^ ^50^ ^]^ Due to the electromagnetic induction, a voltage profile (EMF) will then be produced in the conductive coil according to the micropillar vibration. Through the time‐to‐frequency transformation, the frequency characteristics of the micropillar's oscillation could be determined. In principle, this eigenfrequency is mainly defined by the micropillar, which allows us to customize the MMPs for specific eigenfrequency production and command allocation. Details about the signal process of time to frequency domain conversion (Prony method) were discussed in Note S1 (Supporting Information). As depicted in Figure 1g, even though three micropillars have been integrated onto the same coil substrate with one communication channel, the vibration can produce distinguishable electrical signals. The oscillating signals can be converted to the non‐overlapping signals in frequency domain, and thus the pillar‐based eigenfrequency can be applied as an effective and accurate solution for the specific command allocation. In principle, with more integrated MMPs, the generation of specific eigenfrequencies can be allocated for more commands to build a multifunctional interface with one communication channel.

### 2.2 Characterization and Authentication

**Figure 2:**
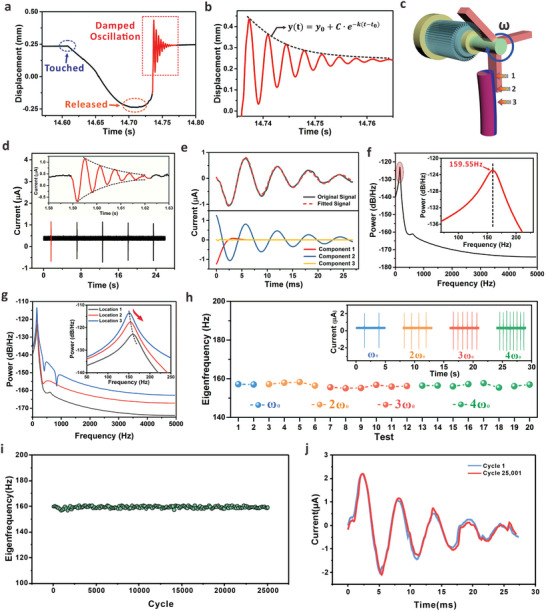
Characterization of eigenfrequency performance. a) Oscillation of MMP measured by LDV. The MMP was manually vibrated via a tweezer, and damped oscillation was observed once the mechanical constrain was released. b) Enlarged profile of the damped oscillation after the removal of external force. The curvature was fitted by an exponential decay function. c) Schematic diagram of the experimental setup for standard characterization of the MMP eigenfrequency. The locations of 1, 2, and 3 can be adjusted according to the relative displacement between the blade and the MMP. Speed of the blade can be controlled by the motor. d) Record of the electrical current during five cycles’ periodical vibration of the MMP. The red curve in the inset shows the enlarged real‐time profile of the current in a damped oscillating form. e) Fitting of the current signals and corresponding components after the signal processing based on Prony Method analysis. f) Plot of the power spectrum dependent on the frequency domain. The eigenfrequency with maximum power allocation was indicated in the inset. g) Electrical response of the MMP when exposed to the blade impact at three different locations. h) Stability investigation of the produced eigenfrequencies with different impact speeds on the same MMP. i) Fatigue test of the micropillar H5.0P0.5E0.5 with corresponding eigenfrequencies for 25 000 cycles. For a better observation, we selected one typical point to represent the behavior for every 150 data points of the eigenfrequency. j) Electrical signals of the first cycle and 25001 cycles during the fatigue test.

To characterize the real‐time vibration of the micropillar, the Laser Doppler Vibrometer (LDV) was employed to track the displacement of MMP throughout the process. We applied a tweezer to deform the free end of a typical MMP (H4.0P0.5E0.5), and the laser spot of LDV was focused at the free end to track the instantaneous displacement. Here, H4.0P0.5E0.5 indicates that the height of the MMP is 4 mm, and the polymer matrix consists of 50% PDMS and 50% Ecoflex in mass ratio. Note that the radius (*R*) of all MMPs in this work is fixed at 0.5 mm. The entire process is presented in **Figure** **2**a. When touched by the tweezer, a negative displacement was recorded at the free end of the micropillar, which finally reached the maximum negative value once the mechanical constrain was released. After that, an inherent recovery was quickly observed on the MMP, and the enlarged waveform of inherent damped oscillation is presented in Figure 2b. An exponential decay function (OriginLab) was adopted to fit the decay of the discrete displacement peaks in the waveform, with a fitting results of $y\;\left(t\right)=\;0.2349+0.2\cdot e^{-\frac{t-14.737}{0.0078}}$ (Figure S4a, Supporting Information, R^2^ coefficient of 0.9998). With time advancing, the intrinsic oscillating displacement gradually decreased, and finally vanished after several cycles in ≈26.9 ms. The results show that the inherent oscillation of MMP is following the waveform of damped sinusoid. In addition, we used LDV to record the dynamic velocity of MMP in a typical oscillation process, which also exhibits a periodical oscillation in damped sinusoidal behavior (Figure S4b, Supporting Information). As the flexible micropillar has been magnetized, the localized magnetic field would vary periodically to induce electrical currents in the coil underneath. Figure 2c shows the schematic diagram to vibrate the MMP for measurement of the electrical current, and the optical image of the entire setup is displayed in Figure S5 (Supporting Information). As discussed below, the impact location on the micropillar can be flexibly adjusted to investigate the deformation influence, while the rotation speed of the blade was applied to consider the effect from the external impact. Via vibrating the micropillar (H5.0P0.5E0.5) for five consecutive times, the typical induced electrical currents within the coil was shown in Figure 2d. The red curve in the inset shows the enlarged real‐time profile of the current in a damping mode, which is mainly caused by the recovery and periodical oscillation of the micropillar. The characteristics of the induced current basically follow the damped behavior of the free‐end motion (displacement) as shown in Figure 2a,b, which ensures that the electromagnetic induction is also an effective approach to reflect the vibration process. To explore the oscillation frequency that is related with a specific MMP, the conversion from time to frequency domain was performed on the induced current. A nonlinear fitting (OriginLab) was first introduced to process the original signal, and three main components in damped sinusoidal form, $y\;\left(t\right)=A_{i}\;e^{\sigma_{i}t}\cos\left(2\pi f_{i}t+\theta_{i}\right)$, were shown in Figure 2e. Here, *A_i_* is the related amplitude, *f_i_* is the sampling frequency, and *θ_i_* is the phase constant. Note that all the signals were shifted down to cancel the noise from the electrical instrument before the data processing. Table S1 (Supporting Information) provides the details of fitting parameters, and the Prony method for conversion from time to frequency domain is explained in details in Note S1 (Supporting Information). In principle, the oscillation eigenfrequency is determined by the inherent property, e.g. the dimension, and the mechanical strength, of the MMP. The Prony energy spectrum (Figure 2f) shows a sharp peak at ≈159.55 Hz across the whole frequency domain, which can be considered as the inherent eigenfrequency of the micropillar (H5.0P0.5E0.5) under the specific conditions. From this perspective, the incorporation of electromagnetic induction into vibrating micropillars can effectively generate the signals to determine the eigenfrequency. As discussed subsequently, the experimentally‐obtained eigenfrequency is consistent with the theoretical model, thus enabling the precise adjustment of the eigenfrequency to realize a frequency‐dominant interactive interface.

Figure S6 (Supporting Information) further compares the eigenfrequencies from the oscillating electrical signals in Figure 2d. The stable generation of frequency signals across different tests ensures the reliability of the proposed mechanism and interface. We notice that for the real applications, the human finger may not be able to touch the MMP at a fixed location due to the small micropillar size (in range of several millimeters). Also, it can be predicted that the customer cannot apply a constant sweeping speed to deform the MMP during the daily uses. Considering this, to demonstrate the influence of the bending angle on the induced current, we adjusted the height of the displacement platform to control the contact position between the blade and MMPs. This will finally drive the MMP to deform with different angles, as shown in Figure S7a (Supporting Information). The first peak value of the current during the oscillation was selected as the characteristic current for comparison, as can be seen in Figure S7b (Supporting Information). As the bending angle gradually increases, the magnitude of the characteristic current induced within the coil increases continuously (Figure S7c, Supporting Information). Further, we performed a series of experiments to illustrate the influence on the recorded eigenfrequency when the external force was applied at different positions of the MMP with various impact velocities. As depicted in Figure 2c, the blade was controlled to impact the micropillars at given locations of 1, 2, and 3, and the corresponding deformations were shown in Figure S8 (Supporting Information). The induced current signals in Figure S9a (Supporting Information) reveal that when the impact position moves downward, the maximum deformation degree increases, resulting in a gradually rising signal amplitude. The corresponding Prony energy spectrum was given in Figure 2g. It can also be observed that the shapes of the frequency spectra are similar, and the peaks of the eigenfrequencies are located almost at the same position (Figure S9b, Supporting Information). The inset further exhibits a slight “right shift” of the eigenfrequency when the impact position was moved up from Location 3 to Location 1. This behavior might be attributed by the damping effect from the air, which imposes a tiny revision to the eigenfrequency. As the parasitic drag of the surrounding medium (air) is proportional to the square of the instantaneous speed, the lower impact position would induce a higher oscillating speed to cause the decay of the eigenfrequency.^[^ ^51^ ^]^ The results confirm that the impact position on the MMP has negligible influence on the eigenfrequency of a given micropillar. We further discussed the influence on the eigenfrequency when the impact velocity of the blade was changed. The vibrating velocity could be flexibly tuned by changing the motor speed. As shown in Figure S5 (Supporting Information), the motor speed was adjusted from ω_0_ to 4ω_0_ (ω_0_ is 10% of the speed upper limit) to impact the micropillar at the same position. Details of the induced current based on different vibrating speeds are provided in Figure S10 (Supporting Information). It is clear that the electrical signals related with the micropillar deformation are tightly dependent on the impact velocity (ω_0_ and 4ω_0_), and a higher impact velocity results in a faster signal generation (from 70.2 to 16.8 ms). However, the measured eigenfrequencies kept almost the same, indicating the oscillation frequency is the inherent property of the studied micropillar. Figure 2h summarizes the influence of motor speed on eigenfrequencies. The eigenfrequencies remained almost unaffected by the impact speed from the blade. Furthermore, the reliability and robustness of the interface were studied by randomly locating the MMP (H5.0P0.5E0.5) at the left/right edge or the central region of the coil. The corresponding electrical signals were recorded and compared as shown in Figure S11 (Supporting Information). The intensity variation of the induced current was attributed by the amount of magnetic flux that is location‐dependent (Figure S11a, Supporting Information). However, the eigenfrequency, which was mainly determined by the micropillar, remains almost identical without obvious interference from the position on the coil (Figure S11b, Supporting Information). Figure 2i further shows the long‐term stability of the eigenfrequency when the MMP (H5.0P0.5E0.5) was exposed to cyclic deformation for 25 000 times. For a better observation of the stability performance, a single point was randomly selected from every 150 data points. The eigenfrequency maintained ≈160 Hz for the whole test, and no obvious variation of the eigenfrequency values was observed after the fatigue test, indicating the robustness of the micro‐scaled MMP as a reliable interface for practical wearable interactions. Further, Figure 2j illustrates the characteristics of the 1st and 25 001th oscillation signals in time domain, from which we can observe that the two signals almost overlapped in a similar response behavior. It can be expected that the flexible and elastic MMP can withstand the mechanical deformation that is induced by the human finger under daily conditions.

### 2.3 Customization of Eigenfrequencies

**Figure 3:**
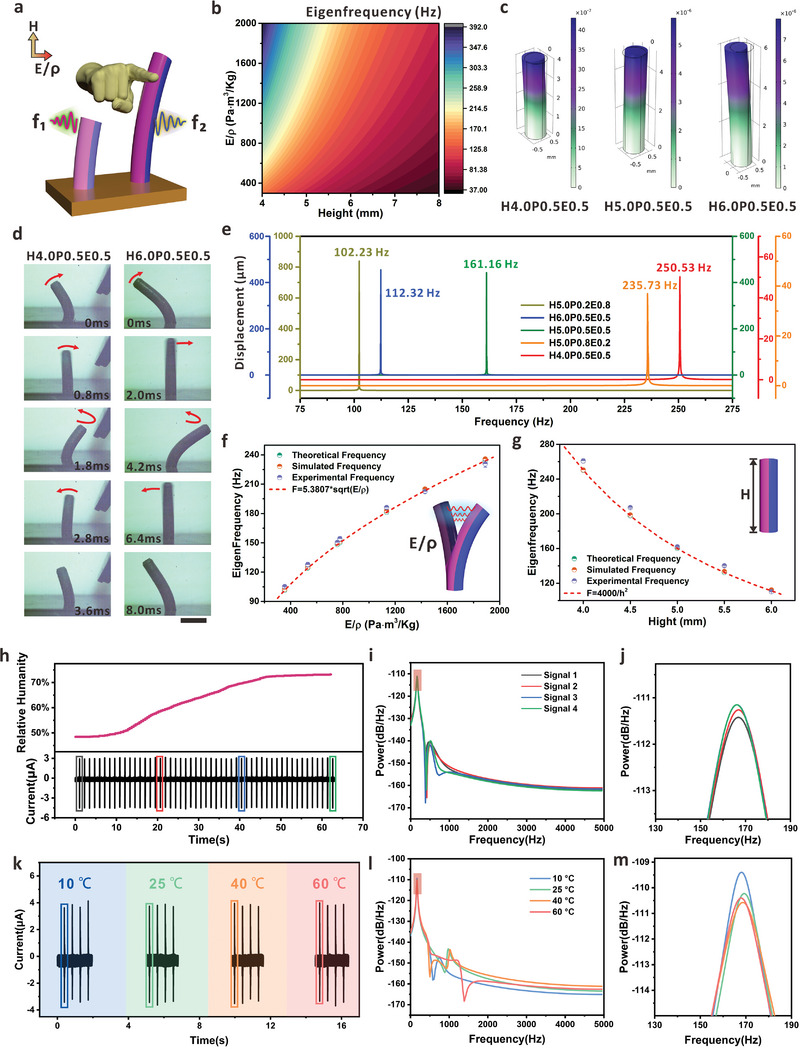
Customized eigenfrequencies of the MMP. a) Schematic diagram of eigenfrequency control via dimension and modulus/density of the MMP. The color indicates the modification of the material property of the micropillar. b) Theoretical eigenfrequency of the MMP with variables of height and modulus/density. c) Simulation of the eigenfrequency from MMPs with different parameters. The surface contour is displacement magnitude with unit of *mm*. d) Simulation result of displacement under external force with different frequencies. The maximum displacement indicates the corresponding frequency is the resonance and also the eigenfrequency of the micropillar. e) Real‐time vibration record from the MMP of H4.0P0.5E0.5 (left) and H6.0P0.5E0.5 (right). The scale bar is 2 mm for all optical images. f) Regulation of eigenfrequency based on different values of *E/ρ* from the MMPs. g) Regulation of eigenfrequency via tuning the height (*H*) of the MMPs. h) Oscillation signals of H5.0P0.5E0.5 in different humidity conditions. i) Prony energy spectrum of 4 signals marked in (h). j) Enlarged profile of Prony energy spectrum around the characteristic peak. k) Current signals of H5.0P0.5E0.5 oscillation in 4 different temperature conditions. l) Prony energy spectrum of 4 signals marked in (l). m) Enlarged profile of Prony energy spectrum around the characteristic peak.

For a reliable, multifunctional, and effective HMI, it's crucial to generate identifiable eigenfrequencies so that the customized commands can be allocated to the specific micropillars. Considering this, a cylindrical cantilever model was adopted to investigate the parameters that can possibly tune the eigenfrequency of the MMP. The schematic diagram exhibits that via tuning the dimension (height, *H*) and material property (modulus/density, *E/ρ*) of the MMP, the related eigenfrequency (*f_1_* and *f_2_*) can be possibly regulated (**Figure** **3**a). Based on the specific eigenfrequency design, the micropillar array with controllable eigenfrequency can thus enable a multi‐functional interface for interactions, which is established on one coil device. For example, when the electrical terminal receives the frequency of *f_1_*, a command can be performed, while another frequency of *f_2_* can trigger another pre‐encoded command without interference. Figure 3b illustrates the theoretical eigenfrequency of the MMP based on the combinational variables of height (*H*) and modulus/density (*E/ρ*). The depth of the micro‐holes in the mold determines the height of the MMP, while the modulus and density of the MMP can be regulated via changing the mass ratio between PDMS and Ecoflex (Figure S12 and Table S2, Supporting Information). According to the cylindrical cantilever model, the relationship between eigenfrequency (*f*) and related parameters is given by:

(1) $$f\;=\frac{1.875^{2}}{4\pi H^{2}}\;\sqrt{\frac{ER^{2}}{\rho }}$$

where *H* is the height of the MMP, *E* is elastic modulus, *R* is radius of cross‐sectional circle, and *ρ* is the material density (see Note S2, Supporting Information for the detailed derivation process). With a fixed radius, *R*, of 0.5 mm, the relationship was finalized as:

(2) $$f\;=\;\left(5\times 10^{-4}\times \frac{1.875^{2}}{4\pi }\right)\cdot \frac{1}{H^{2}}\cdot \sqrt{\frac{E}{\rho }}$$

Based on the above formula, the theoretical relationship between the eigenfrequency (*f*), the height (*H*), and the material property (*E/ρ*) is presented in Figure 3b. Normally, a smaller *H* or a larger *E/ρ* can both result in the increase of the eigenfrequency. The FEM (finite element method) simulation results in Figure 3c further indicate that the eigenfrequency is dependent on the micropillar properties. When a horizontal force was applied to the micropillar, the lateral displacement of the free end continuously increases as the height changes from 4 to 6 mm. The eigenfrequencies also exhibit the decay from 250.63 Hz, 161.16 Hz, to 112.32 Hz, which is in consistence with the theoretical values in Figure 3b.

Figure S13 (Supporting Information) further provides the simulation results of the eigenfrequencies based on different micropillar heights and modulus/density. The simulation in frequency domain records the resonant frequency of different micropillars, which can be considered as the eigenfrequency when the maximum displacement peak was observed (Experimental Section provides more details about the simulation setting). As shown in Figure 3d, the typical eigenfrequencies are obviously dependent on the height and the modulus/density of the micropillars. For example, with fixed height of 5 mm, the eigenfrequency of the MMP (H5.0P0.8E0.2, H5.0P0.5E0.5, and H5.0P0.2E0.8) exhibits obvious decay according to the decrease of *E/ρ* (Figure S12d, Supporting Information). To further confirm the frequency difference, a high‐speed camera was used to record the oscillating behavior of the MMPs within one whole cycle (Figure 3e; Video S1, Supporting Information). As shown in the snapshots, one oscillation period is roughly 3.6 ms for H4P0.5E0.5 and ≈8.0 ms for H6P0.5E0.5, which well matches the theoretical and simulated eigenfrequencies. To experimentally exam the eigenfrequency, we tuned the height (*H*) and the modulus/density (*E/ρ*) of the micropillar and the induced currents in the coil were recorded as shown in Figures S14 and S15 (Supporting Information). The electrical signals were processed based on the Prony method to figure out the corresponding eigenfrequencies which are mainly determined by the intrinsic properties of the MMP. Figure 3f compares the values of the MMP eigenfrequency with different *E/ρ* while maintaining the *H* at 5 mm. The results match well and the fitted curve, $f\;=\;5.3807\sqrt{\frac{E}{\rho }},$ is the theoretical profile that expresses the possible regulation of eigenfrequency via tuning the value of *E/ρ*. Similarly, Figure 3g shows the experimental, theoretical, and simulated eigenfrequency of MMP for different heights from 4 mm to 6 mm, while keeping the *E/ρ* at 777.6 Pa·m^3^ Kg^−1^ (related with case of P0.5E0.5 as shown in Figure S12d, Supporting Information). The dashed line shows the fitted curve, $=\frac{4000}{h^{2}}\;$, which provides the guideline to tune the eigenfrequency via the dimensional parameter of the micropillar. From the theoretical analysis to experimental proof, the results confirm that the MMP eigenfrequency can be flexibly controlled through the property regulation, thus providing a possible solution to generate multiple commands for effective human–machine interactions. Note that the micropillars can share the same conductive coil for signal communication, and the necessity to increase the burden on wiring or device spacing can thus be completely avoided. Consequently, the eigenfrequency‐dominant approach is capable to produce multiple commands in an interference‐free manner even based on one communication channel. To demonstrate the oscillation properties of MMPs across various environments, the device and whole testing platform were deposited in different humidity and temperature environments (see Figure S16, Supporting Information for the experimental setups). As shown in Figure 3**h**, the relative humidity of the environment was adjusted from 48.4% to 73.2% in 62 s. The induced current within coil corresponding to oscillation of H5.0P0.5E0.5 was recorded simultaneously. Four oscillation signals under different humidities were selected for comparative analysis, and the Prony energy spectra were demonstrated in Figure 3i (see Figure 3j for enlarged profile near the characteristic peaks). Notably, the eigenfrequencies of four signals remain identical, signifying the robustness of MMP under different humidity conditions. Similarly, we placed the device on a hot plate and a semiconductor cooler, respectively, to compare the electrical signals in different temperature environments. The signals in time domain under four temperatures (10, 25, 40, and 60 °C) are shown in Figure 3k. Figure 3l,m demonstrate the Prony energy spectra of the signals at the four temperatures, and the similar positions of eigen‐peaks indicate that the eigenfrequencies do not change obviously with the temperatures. The MMPs exhibit excellent robustness and durability in various conditions, which is capable for applications in different scenarios.

### 2.4 Demonstration of Eigenfrequency‐Dominant High‐Capacity HMI

**Figure 4:**
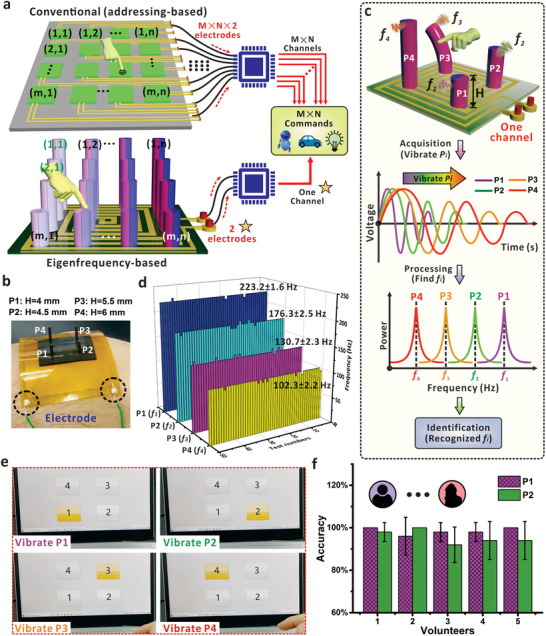
Verification of eigenfrequency‐based HMI systems. a) Comparison of HMI systems using conventional addressing‐based and eigenfrequency‐based approaches. To realize command capacity of m × n, m × n × 2 electrodes and m × n channels are required for addressing‐based method, while two electrodes and one channel are required for eigenfrequency‐based method. b) Optical image of the wearable interface with four integrated MMPs and one coil substrate. c) Schematic diagram of customized HMI systems based on four MMPs with different heights and thus the eigenfrequencies. The eigenfrequencies were encoded with different commands to realize high‐capacity HMI. d) Frequency stability corresponding to 50 cycles’ consecutive vibration of the MMPs. Each MMP was vibrated 50 times, and the collected vibration signals were analyzed in frequency domain. e) Identification of the mechanical inputs by MMP‐based HMI system. By evaluating the eigenfrequency of the vibration signals, position distribution of the input signals could be obtained. f) Robustness test for MMP system. Five volunteers were involved to continuously vibrate P1 and P2 for 50 times and the accuracy was recorded.

**Figure** **4**a provides the comparison between the conventional and eigenfrequency‐based high‐capacity HMI system. Normally, the electrical channels of *m* × *n* are required for conventional interface to establish a command capacity of *m* × *n*.^[^ ^52^, ^53^ ^]^ When the mechanical input was applied to one specific sensor, the corresponding channel received the electrical pulse and the pre‐set operation or command would be delivered to the terminal. Consequently, this addressing‐based approach normally requires the electrode amount of *m* × *n* × 2 to avoid interference, which will bring complexity to the interface with increased values of *m* and *n*. As discussed above, the eigenfrequency of MMP can be customized to build‐up the interaction system that is mainly based on the differential frequency values. From this perspective, only two connecting electrodes are required for eigenfrequency‐based HMI interface, which is independent of the array number of *m* and *n*. For example, if 16 commands are necessary for a specific HMI interface, 16 communication channels and 32 connecting electrodes should be applied for conventional sensor arrays. However, only one communication channel and two connecting electrodes are required for the eigenfrequency‐based interface. As a proof of concept, we first deposited four typical MMPs (P1‐H4.0P0.5E0.5, P2‐H4.5P0.5E0.5, P3‐H5.5P0.5E0.5, and P4‐H6.0P0.5E0.5) on one coil substrate to establish the HMI system. The design of these MMPs will generate four different eigenfrequencies, *f_1_*, *f_2_*, *f_3_*, and *f_4_*, that present the progressive decrease owing to the increased height from 4.0 to 6.0 mm. The optical image (Figure 4b) shows that the four MMPs can be integrated to build up a multifunctional HMI interface with two electrode connections. When a typical MMP was vibrated by the human finger, the coil underneath would perceive the magnetic field variation that is related with the micropillar oscillation. As shown in Figure 4c, the one‐output terminal can collect the signals from the vibration of the MMP, and convert to individual frequency for the interaction with multi‐commands. In principle, not only the height but also the mechanical property of the MMPs can be applied to tune the eigenfrequency if more commands are required.

To illustrate the reliability of the interface, each MMP was continuously vibrated for 50 times by manual operation and the frequency of MMPs’ oscillations was recorded by a LabVIEW‐based script (Figure S17, Supporting Information). As presented in Figure 4d, the finger sweeping on P1 produced an average eigenfrequency at 223.2 Hz, with a narrow derivation of ±1.6 Hz. Eigenfrequencies of other MMPs are recorded as 176.3±2.5 Hz, 130.7±2.3 Hz, and 102.3±2.2 Hz, respectively. The non‐overlapping behavior confirms the eigenfrequency‐based approach as a reliable recognition mechanism for command encoding. As demonstrated in Figure 4e, the vibration of specific MMPs can be perceived and lighten the lamps of 1–4 (Video S2, Supporting Information). When an MMP with a particular frequency is vibrated by a finger, the electrical signal was induced and the corresponding eigenfrequency could be determined by the software in the computer terminal, which finally was reflected by the highlighted number as displayed. To further investigate the interface reliability for identification of the input source, five volunteers participated in the test to successively vibrate P1 and P2 for ten times and the accuracy was summarized in Figure 4f. As shown in Video S3 (Supporting Information), the two MMPs were identified with accuracy of over 90%, showing that the eigenfrequency‐based mechanism is of reliable potential in the daily human–machine interactions toward a broad spectrum of users.

**Figure 5:**
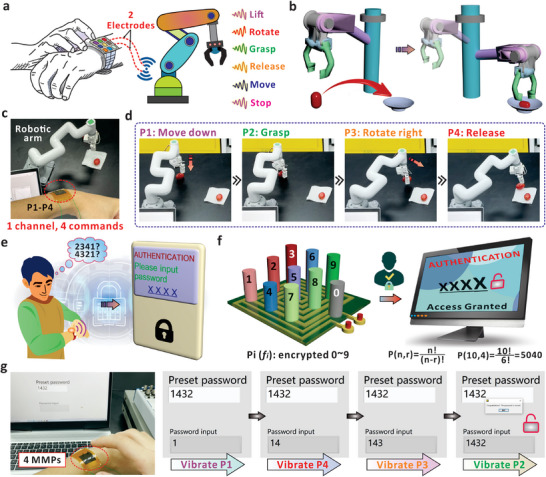
Four MMPs‐based HMI interface. a) Schematic diagram of customized HMI for intelligent robotic control. Two electrodes are required in the system owing to the specific eigenfrequencies from different micropillars. b) Illustration of transferring the object from one to another location via robotic operation. c) Optical image of the robotic arm under control of the MMPs‐based wearable interface. d) Process of transferring the tomato target via robotic arm based on the developed interface. The four MMPs are related with the commands of “Move down”, “Grasp”, “Rotate right”, and “Release”. e) Schematic diagram of the authentication system based on the wearable HMI interface. f) Illustration of MMP‐based password recognition system. With ten MMPs, the numbers of 0–9 can be allocated to ensure a complete authentication interface. g) Record of authentication process based on four MMPs which are related with the numbers of 1, 2, 3, and 4. The password is pre‐defined in the software and the user inputs the digits by vibrating the MMPs in sequence.

Based on four MMPs design, we demonstrated that the assembly could be applied to produce different allocated commands for effective robotic arm control and the authentication system. The schematic diagram in **Figure** **5**a shows that the integration of multiple MMPs is capable to produce different commands of “Lift”, “Rotate”, and “Grasp”, etc. With two electrodes and one channel, the distinguishable eigenfrequencies can be used to allocate the specific command with accuracy. For robotic control application, we show that the four MMPs system is effective to move the target from one to another location (Figure 5b). As shown in Figure 5c, the experimental setup consists of the wearable interface (4 MMPs and the coil), the computer system, and the robotic arm. The oscillation of the MMPs were captured by the coil beneath and transmitted to the software script for frequency determination, then a command was sent to the robotic arm to perform the corresponding operation that has been encoded. When P1 was vibrated, the interface received the corresponding eigenfrequency signal, and the pre‐defined command of “Move down” was transmitted to the robotic arm. A “move down” action was performed by the robotic arm to approach the tomato target (Figure 5d). When P2, P3, and P4 were vibrated successively, corresponding commands, e.g. to grasp the tomato, to rotate right, and release the tomato, were conducted on demand. As shown in Video S4 (Supporting Information), this entire interaction process requires only one MMPs‐based interface to achieve the multi‐commands, instead of using the device array with complex wiring connections. We further demonstrated that the MMPs‐based interface could be applied for effective password coding and decoding in wearable HMI (Figure 5e). To improve the security level, it is inevitable to introduce more digits, which imposes the concern of wiring complexity for the entire system. Alternatively, the eigenfrequency‐based mechanism allows the establishment of authentication in a more effective manner. For example, a total amount of 5040 password combinations can be generated if ten MMPs were integrated and four different digits were selected for permutation (Figure 5f). Note that the interface uses the eigenfrequency for number identification (0–9) and only one channel is required to connect the mechanical input and the electric terminal. Figure 5g shows the process of password inputting and unlocking based on our proposed system by means of eigenfrequency using four MMPs. First, a password (“1432”) is pre‐defined in the software and the MMP interface is used as a numeric keypad. When an MMP is vibrated, the corresponding number would be typed into the input window based on the received eigenfrequency. If the vibration sequence matches the pre‐defined password, the software could detect the inputs, and the system was unlocked successfully. The vibration sequence of P1, P4, P3, and P2 results in the input of 1‐4‐3‐2 to unlock the software interface (Video S5, Supporting Information). Figure S18 (Supporting Information) also shows that via re‐defining the sequence of micropillar vibrations, another password (“4123”) can be encoded through the interface. The vibration order of P4, P1, P2, and P3 was thus applied to realize the unlocking function. From this perspective, a password setting with higher confidentiality, e.g. more digits, can be accessible based on more MMPs with different eigenfrequencies. The design would not bring the wiring or space burden to the entire interface, because the designed micropillars can be integrated onto one coil and only one electrical output was required to enable the recognition process.

**Figure 6:**
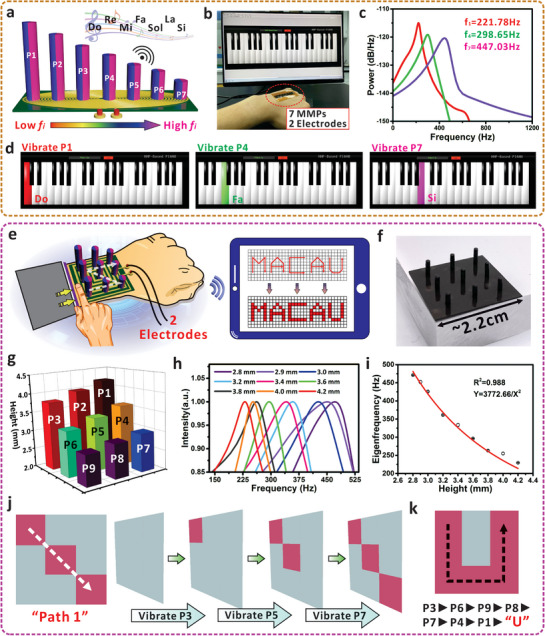
MMPs‐based interface with higher capacity for entertainment and trajectory monitoring. a) Schematic diagram of HMI interface for generation of musical tones based on seven MMPs. b) Optical image of the wearable device and software interface for demonstration. Two electrodes are used and seven tones can be completely produced with 7 MMPs. c) The measured eigenfrequencies of micropillars P1, P4, and P7 used in the demonstration. d) Generation of musical tones “Do”, “Fa”, and “Si” via the oscillation of P1, P4, and P7. e) Schematic diagram of the wearable interface for trajectory monitoring based on MMPs. f) Optical image of a 3 × 3 MMPs array for demonstration of trajectory tracking. g) Design of the height gradient in a continuously decreased behavior from P1 to P9. h) Relationship between the normalized electrical intensity and the frequency based on MMPs with different heights. i) Fitting results and relationship between the measured eigenfrequency and the height of the MMPs. j) Trajectory tracking of a diagonal path via deforming the micropillars P3, P5, and P7 in sequence. k) Generation of letter “U” via combinational vibration of P3, P6, P9, P8, P7, P4, and P1.

As mentioned above, the increased demand of command capacity would bring the complexity of wiring system or electrode connections, especially for the cases when values of *m* and *n* are becoming larger. However, the eigenfrequency‐based approach can effectively avoid the limitation and two electrodes are normally required even with more MMPs in the same system. **Figure** **6**a,b present the demonstration of a wearable electronic piano that can produce seven musical tones based on different MMPs. Within an identical coil, seven MMPs (P1‐P7) with heights of 4.0, 3.8, 3.6, 3.4, 3.2, 3.0, and 2.8 mm were integrated and each micropillar represents a specific tone from “Do” to “Si”. The MMPs were integrated in a row (7 × 1), and arranged in the form of continuously increased eigenfrequency from P1 to P7. As shown in Figure 6c, the recorded eigenfrequencies of P1, P4, and P7 are 221.78, 298.65, and 447.03 Hz, respectively. The non‐overlapping behavior of eigenfrequencies allows the users to allocate specific musical tones to the micropillars instead of using a wiring system for location addressing.^[^ ^54^, ^55^ ^]^ For example, the related keys of “Do”, “Fa”, and “Si” were recognized when the micropillars P1, P4, and P7 were manually deformed (Figure 6d; Figure S19, Supporting Information). Video S6 (Supporting Information) also records the complete process to produce musical tones from “Do” to “Si”, which can be easily performed based on the wearable interface that has been integrated with seven MMPs of different eigenfrequencies. Furthermore, the vibration of MMPs can be potentially applied to reflect the motion trajectory as each micropillar can serve as a specific location that can be identified via the non‐overlapping eigenfrequencies.^[^ ^47^, ^56^ ^]^ When a specific eigenfrequency is received, the related location can be determined and the continuous deformation of the micropillars can be combined to reflect the trajectory from different pixels, such as “MACAU” (Figure 6e). As a proof of concept, a 3 × 3 MMPs array was fabricated to exhibit the potential for trajectory mapping and interactive writing. Figure 6f shows the assembly of nine MMPs with a total area of ≈2.2 cm × 2.2 cm, which was then assembled with the coil for HMI demonstration (Figure S20a,b, Supporting Information). The heights of the MMPs in Figure 6g were designed from 4.2 mm (P1) to 2.8 mm (P9), and the related eigenfrequencies were recorded as shown in the normalized spectrum in Figure 6h. With increased height, the eigenfrequency continuously decreases from 471.25±0.80 Hz to 229.55±0.84 Hz. The frequency trend follows the governing formula of *f*∝*H* ^−2^, where a larger MMP height (H) would lead to the decreased eigenfrequency in exponential behavior (Figure 6i). With the non‐overlapping eigenfrequencies, it is thus possible to identify the positions of mechanical inputs and convert to the real‐time trajectory. Figure 6j displays the vibration path of P3, P5, and P7 can be applied to represent the diagonal trajectory of Path 1, and a letter “U” was successfully realized with a more complex triggering path of P3, P6, P9, P8, P7, P4, and P1 (Figure 6k). Another path (Path 2) and letter “C” were also provided in Figure S20c (Supporting Information) through the combinational oscillation of different MMPs, and the complete interaction process was recorded in Video S7 (Supporting Information). With design of more MMPs, we believe that the eigenfrequency‐based mechanism can provide the possibility to realize more functions for further HMI, while without imposing the burden on the overall system consumption and the number of connecting electrodes.

## 3 Conclusion

In this work, we reported a wearable HMI interface which uses the regulation of eigenfrequency as the dominant perception mechanism. When the MMPs were deformed, the intrinsic oscillation caused the variation of the magnetic field distribution and the instant current was received in the conductive coil. By converting the signals from time to frequency domain, the eigenfrequency that is mainly determined by the micropillars could be identified as the marker for subsequent coding and decoding. Along with the theoretical model and simulation results, we experimentally proved that the eigenfrequency can be flexibly customized via the material property and dimension of the micropillars. Based on one coil device, we built up a conceptual interactive platform which consists of four MMPs with different heights and thus the eigenfrequencies. The platform could conveniently produce multiple instructions through the intrinsic oscillation of specific MMPs, exhibiting the potential for uses in robotic arm control and password recognition system. With more MMPs, the non‐overlapping eigenfrequencies allow us to realize the electronic piano or the trajectory mapping/handwriting on the platform using one electrical channel. The demonstrations confirm the improved capacity and functionalities would not bring the concern of electrode numbers or connection system that might affect the wearability. Thanks to the robustness of the MMP, the interference‐free eigenfrequency generation also verifies the developed interface can be a reliable and accurate medium for daily applications. We believe that the design of eigenfrequency‐based HMI device could be more effective to address the continuously increasing demand of multiple commands for the next‐generation HMI and IoT era.

## 4 Experimental Section

### Materials

The neodymium‐iron‐boron (NdFeB) particles were purchased from Magnequench, China. Polydimethylsiloxane (PDMS) base and curing agent (Sylgard 184 kit) were purchased from Dow Corning, USA. Ecoflex (model of 00–50) was from Smooth‐On, Inc, USA. The substrate of copper coil was purchased from Chengdu Do‐itc New Material Co., Ltd. (China), and subsequently processed via laser machine for coil pattern formation.

### Fabrication of MMP and Conductive Coil

Laser patterning of the copper coil was conducted with laser engraving machine (LPKF ProtoLaser U4, LPKF Laser& Electronics AG, Germany). The width of each loop is ≈70 µm and the distance between two adjacent conductive lines is ≈80 µm. The coil consists of two layers, which are separated by an insulating polyimide film, and each layer contains 50 turns. After engraving, the coil was bathed in citric acid to remove the oxidized layer, and a drop of conductive silver glue was deposited on the hole to connect both layers. Finally, a plastic capsulation was applied to the coil to avoid oxidation during the use. For the MMP, a plastic Polymethylmethacrylate (PMMA) mold with pre‐designed micro‐hole array was used as the template for the micropillar (NdFeB/PDMS/Ecoflex) preparation. The mold was fabricated using the engraving machine (CNC‐3020, JingYan Instruments& Technology Co.). First, the Polydimethylsiloxane (PDMS) gel, Ecoflex, and NdFeB particles were uniformly mixed with a specific mass ratio, and the composite was poured into the plastic mold and cured on the hot plate at 80 °C for 30 min to ensure complete solidification. After solidification, the cured PDMS/Ecoflex/NdFeB composite was peeled off from the mold, and placed in the magnetic field with strength of ≈3 T and in‐plane orientations for magnetization. Once magnetized, the MMP sample was stuck onto the copper coil after surface‐treated by plasma cleaner (Harrick Plasma, USA), which helps to improve the interfacial adhesion.

For the MMPs used in the demonstration of robotic control and password setting, the plastic mold was first fabricated with four micro‐holes (radius of 0.5 mm, distance of 20 mm). The micro‐holes with different depths were drilled on the PMMA board (5 cm×5 cm×1 cm) using a milling cutter (radius of 0.5 mm). The depths of the four holes are 4, 4.5, 5.5, and 6 mm, respectively. Then, Ecoflex, PDMS, and NdFeB particles were mixed uniformly with the mass ratio of 1:1:4, and poured onto the prepared mold. The total assembly was then placed in the vacuum chamber to remove the gas that had been trapped in the micro‐holes. After 10 min, the assembly was moved from the vacuum chamber and cured under 80 °C for 30 min. The composite was finally peeled off from the mold for the demonstration. MMPs used in the demonstration of piano playing and trajectory sensing were also fabricated by the similar process. For piano playing, seven micro‐holes (radius of 0.5 mm, distance of 8 mm) were drilled in a row on a plastic template. The depths of the holes are 2.8, 3.0, 3.2, 3.4, 3.6, 3.8, and 4.0 mm. For trajectory sensing, nine micro‐holes (radius of 0.5 mm, distance of 6 mm) were drilled as a 3×3 array on a plastic board. The depths of the holes are 2.8, 2.9, 3.0, 3.2, 3.4, 3.6, 3.8, 4.0, and 4.2 mm. Compositions of the samples for these two demonstrations are the same. Ecoflex, PDMS, and NdFeB particles were mixed uniformly with the mass ratio of 1:1:4. After that, the composite was poured on the prepared mold, then the assembly was placed in the vacuum chamber to remove the gas trapped in the micro‐holes for 10 min. Finally, the assembly was moved to the oven and cured under 80 °C for 30 min to ensure complete solidification. The device was then peeled off from the mold for the demonstration.

### Characterization

The SEM and EDS images were obtained by field‐emission scanning electron microscopy (FE‐SEM, Carl Zeiss, Germany). The hysteresis loops were measured by a physical property measurement system (PPMS) DynaCool instrument (Quantum Design North America, USA) using the vibrating sample magnetometry at room temperature. For standard oscillation test of the MMPs, the assembly was fixed on the platform (Zolix Instruments Co. Ltd., China), and then the MMP was vibrated by a motor (You Maker, China). Meanwhile, the electrical signals generated in the coil were amplified by low noise current preamplifier (MODEL SR570, SRS, USA) and recorded by multifunctional I/O device (USB‐6341, National Instruments, USA). The data were then transmitted to LabVIEW script for analyzing. Real‐time oscillation process was optically recorded by Laser Doppler Vibrometry (VibroOne, Polytec, Inc., USA). For the measurement, a tweezer was manually controlled to deform the micropillar, and the subsequent vibration was recorded. High‐speed camera (VEO 710S, Phantom, USA) was employed to record the oscillation process in a fast‐speed mode. The elastic modulus of the used materials was measured by the commercial motorized platform (ESM303, Mark‐10 Corporation, USA). The wearable demonstrations were carried out by research volunteers with ensured safety and informed consent during the characterization.

### LabVIEW Interface Design for HMI Demonstration

First, the data acquisition parameters were configured (including physical channels, sampling rate, sampling numbers, and trigger conditions, etc.) via the built‐in DAQmx series subVIs of LabVIEW. Since the preamplifier noise at this setting is in range of 0–1 µA, the triggering condition was set at 1 µA. In addition, we set the sampling time as 100 ms because the micropillar oscillation usually lasts for tens of milliseconds. When the acquisition was triggered, data in 100 ms were transmitted to the Spectral Measurement Express VI for spectrum processing and the maximum frequency was extracted using the Tone Measurement Express VI. The outputs of the Tone Measurement Express VI were used as inputs and matched to the pre‐set frequency bands to realize multiple functions for different applications.

### Simulation of Magnetic Field

The software, COMSOL Multiphysics 5.6, was employed to simulate the magnetic field around the MMP. To be consisted with the experiment, a 3D model was adopted. Geometry of MMP was imported with 3D printing files with different dimensions. To simplify the model, the N54 (sintered NdFeB) was given to the micropillars. Air atmosphere was set with the dimension of 10 mm × 10 mm. The mesh was controlled by the physics interfaces with regular size. Magnetic field (no current) module was employed to trace the magnetic scalar potential and magnetic flux density, with the governing equations ***H*** = −∇*V_m_* and ∇ ∙ ***B*** = 0, where ***H*** is the magnetic field vector, ***B*** = *µ*_0_ *µ_r_* ***H*** is the magnetic flux density vector and *V_m_* the magnetic scalar potential. The boundary conditions were set as ***n*** ∙ ***B*** = 0. The initial value of magnetic scalar potential was set as 0. The constitutive relation between the magnetic field and magnetization was governed by ***B*** = *µ*_0_(***H*** + ***M***), where ***M*** is the magnetization vector.

### Simulation of Eigenfrequency

COMSOL Multiphysics 5.6 was further employed to simulate the eigenfrequency of MMP with different dimensional and physical parameters. Solid mechanics and eigenfrequency module were chosen to compute eigenmodes and eigenfrequency of the MMP. Cylinder models with certain dimensions were constructed in built‐in geometry module. PDMS was given to the MMPs, and the material property (density, Young's modulus, etc.) was kept consistent with the experimental results. One end of the cylinder was applied a fixed constrain, and the other end was set at free. To determine the vibration state of the MMP at different frequencies of forces, a boundary load of 0.1 N m^−2^ was applied to different MMPs, and a probe was attached at a location of 3 mm from the fixed end. The displacement at different frequencies of boundary load was recorded. When a maximum displacement was obtained, the applied frequency was considered as the resonant frequency, and thus the eigenfrequency of the investigated MMP in the model was confirmed.

### Statistical Analysis

The data were expressed as the “mean±standard deviation”. Error bars in all figures are the standard deviations obtained from at least five independent measurements unless otherwise stated. All the data were analyzed and performed by Origin Software.

## Conflict of Interest

The authors declare no conflict of interest.

## Author Contributions

S.D. and B.Z. conceived the idea and designed the methodology. B.Z. guided the whole project. S.D. prepared the samples, analyzed the data, and composed the manuscript. Z.D. and S.D. contributed to the circuit design and signal processing. Y.C. fabricated the conductive copper coil. Q.Z., D.Z., and Z.D. participated in the characterization and data collection. All authors discussed the results and commented on the manuscript.

## Supporting information

Supporting Information

Supplemental Video 1

Supplemental Video 2

Supplemental Video 3

Supplemental Video 4

Supplemental Video 5

Supplemental Video 6

Supplemental Video 7

## Data Availability

The data that support the findings of this study are available from the corresponding author upon reasonable request.
