# Supplementary material for: Single Channel Based Interference‐Free and Self‐Powered Human–Machine Interactive Interface Using Eigenfrequency‐Dominant Mechanism
Source: Adv Sci (Weinh). 2024 Jan 29;11(13):2302782. doi: 10.1002/advs.202302782 (PMC10987133; doi:10.1002/advs.202302782)
Supplement: Supplementary file 1 — Supporting Information [file ADVS-11-2302782-s007.pdf]

ADVANCED SCIENCE
Open Access

Supporting Information

for *Adv. Sci.*, DOI 10.1002/advs.202302782

Single Channel Based Interference-Free and Self-Powered Human–Machine Interactive Interface Using Eigenfrequency-Dominant Mechanism

*Sen Ding, Dazhe Zhao, Yongyao Chen, Ziyi Dai, Qian Zhao, Yibo Gao, Junwen Zhong, Jianyi Luo and Bingpu Zhou**

Supplementary Information

Single channel based interference-free and self-powered human-machine interactive interface using eigenfrequency-dominant mechanism

Sen Ding,[1] Dazhe Zhao,[2] Yongyao Chen,[3] Ziyi Dai,[1] Qian Zhao,[1] Yibo Gao,[4] Junwen Zhong,[2] Jianyi Luo,[3] and Bingpu Zhou[1]

[1]Joint Key Laboratory of the Ministry of Education, Institute of Applied Physics and Materials Engineering, University of Macau, Avenida da Universidade, Taipa, Macau 999078, China

[2]Department of Electromechanical Engineering, University of Macau, Avenida da Universidade, Taipa, Macau 999078, China

[3]Research Center of Flexible Sensing Materials and Devices, School of Applied Physics and Materials, Wuyi University, Jiangmen 529020, China

[4]Shenzhen Shineway Technology Corporation, Shenzhen 518000, Guangdong, China

***Corresponding Author.**

Bingpu Zhou, Email: bpzhou@um.edu.mo. Fax: +853-88222426. Tel: +853-88224196.

Keywords: *human-machine interaction, eigenfrequency, damped oscillation, interference-free, self-powered*

Contents

Supplementary Figures..3

Supplementary Tables. 23

Supplementary Videos. 24

Supplementary Note 1. Prony Method for Signal Analysis. 26

Supplementary Note 2. Analysis of cylindrical cantilever vibration. 28

References. 30

Supplementary Figures.

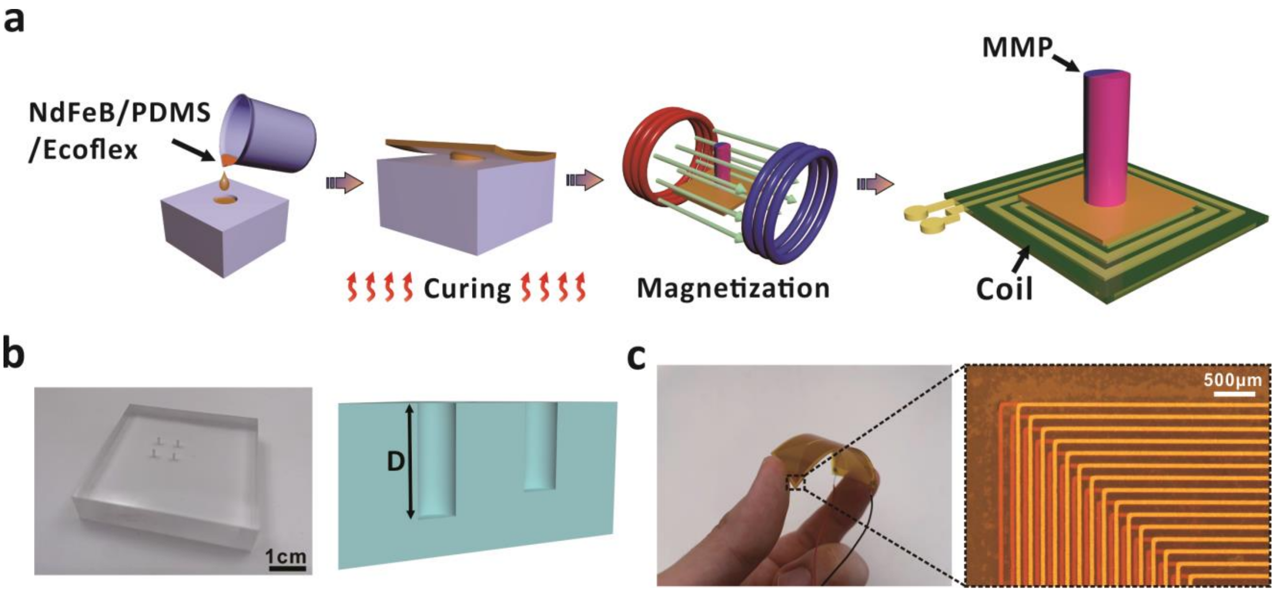

Supplementary Fig. 1. Fabrication process and characterization of the MMP.

a, The fabrication of the human-machine interaction interface based on the combination of conductive coil layer and the magnetized micropillar (MMP). **b**, Optical images of PMMA mold. The schematic diagram shows that the depth (D) of the hole can be tuned to prepare the micropillars with different dimensional parameters. The PMMA mold was prepared with four different depths of 4.0 mm, 4.5 mm, 5.5 mm, and 6.0 mm. Through the casting process, the micropillars with

different heights can be obtained simultaneously. **c**, Optical images of the flexible and conductive copper coil. A two-layered coil layout was used in this work to enable more conductive loops for a higher induced voltage signal. The width of the copper pattern is ~70 μm and the distance between two adjacent conductive lines is ~80 μm.

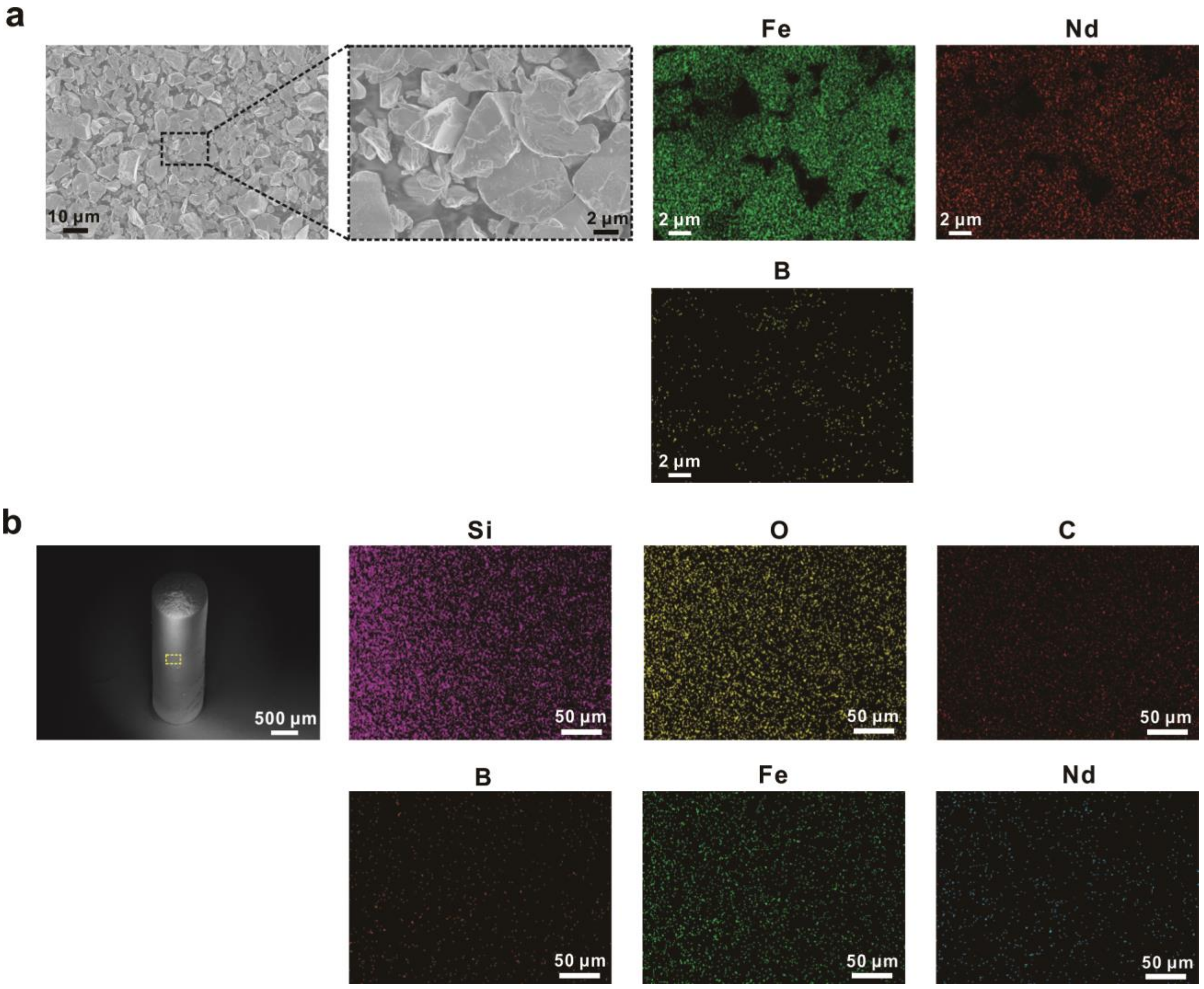

Supplementary Fig. 2. Electron Microscopy (SEM) and Electron Dispersive Spectroscopy (EDS) images.

a, SEM and EDS results of the NdFeB particles. **b**, SEM and EDS images of the micropillar which was prepared by NdFeB, PDMS and Ecoflex.

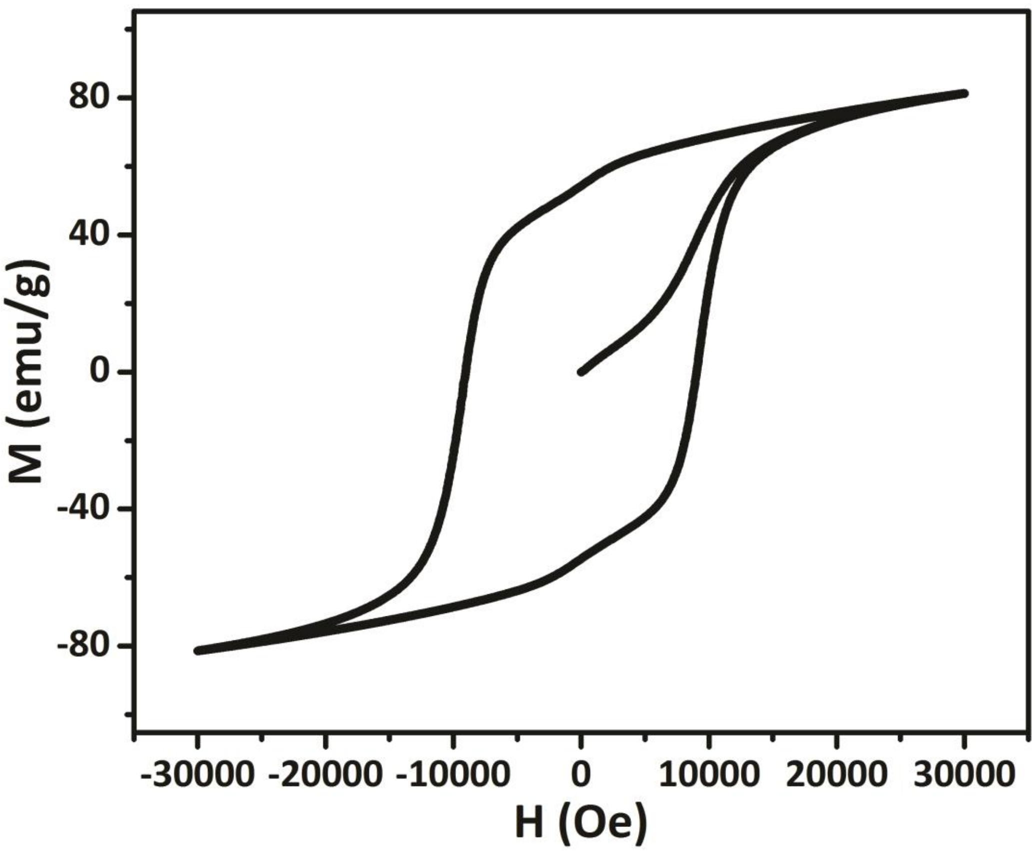

Supplementary Fig. 3. Magnetic hysteresis curvature.

The NdFeB/silicone polymer is prepared by the typical mass ratio of M_{PDMS}:$M_{Ecoflex}$:M_{NdFeB}=1:1:4.

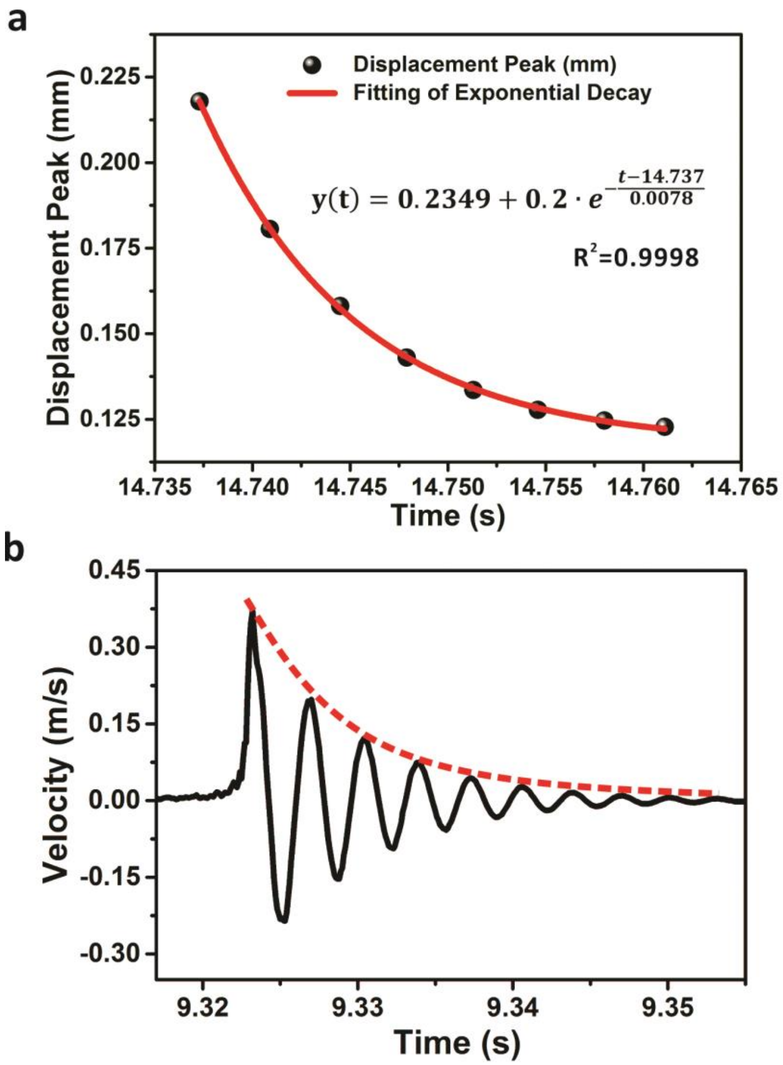

Supplementary Fig. 4. MMP oscillation measurement by LDV.

a, Displacement peak during MMP oscillation shown in **Fig. 2b** and corresponding fitting curvature. **b**, Velocity of one specific point on the micropillar when it has been manually vibrated. The maximum velocity is ~0.4 m/s, and decays with time advancing. Both the displacement and velocity curve exhibit the damped oscillation behavior, which is in consistence with the electrical current signals induced by the magnetic flux variation.

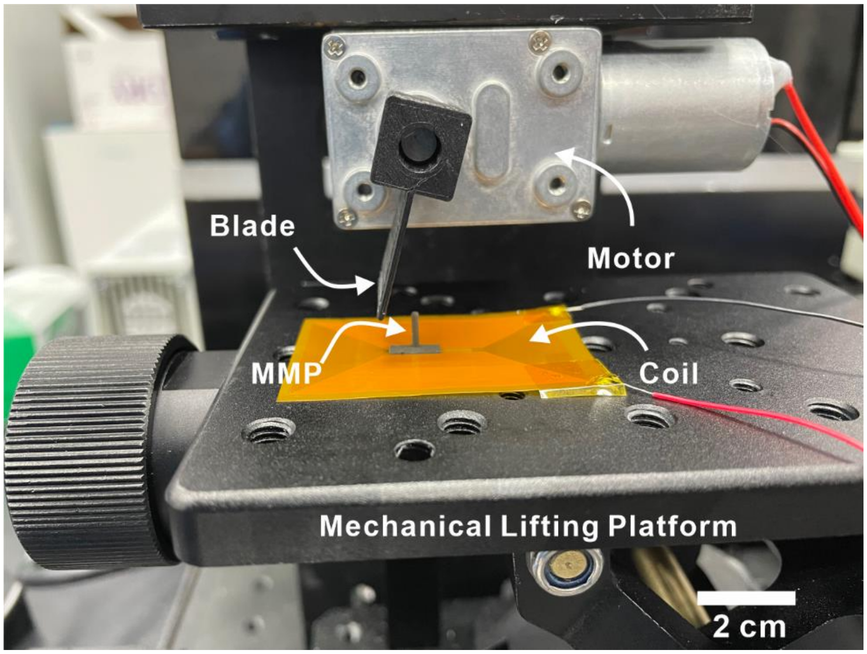

Supplementary Fig. 5. Experimental setup of standard characterization.

The setup consisted of a motor, a blade, and a mechanical lifting platform. The rotation speed of the motor can be changed to vibrate the micropillar with different speeds. The lifting platform is used to tune the relative vertical position of the blade for micropillar deformation. The micropillar was placed on the platform, and the coil was connected with external electrical meter to measure the induced current in real-time.

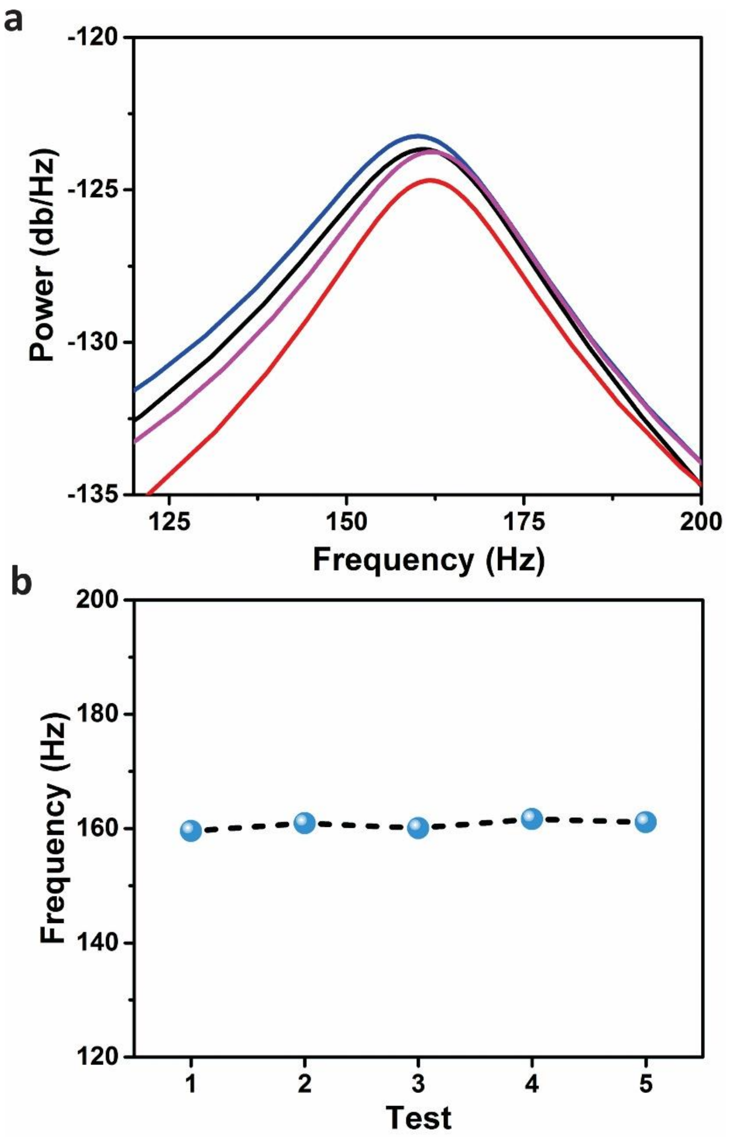

Supplementary Fig. 6. Comparison of signal properties in frequency domain.

a, Prony energy spectrum of current signals shown in **Fig. 2d**. **b**, Comparison of eigenfrequencies corresponding to the five cycles' signals. The variation of the frequency value is insignificant, which confirms the stability and reliability of the frequency-dominant mechanism.

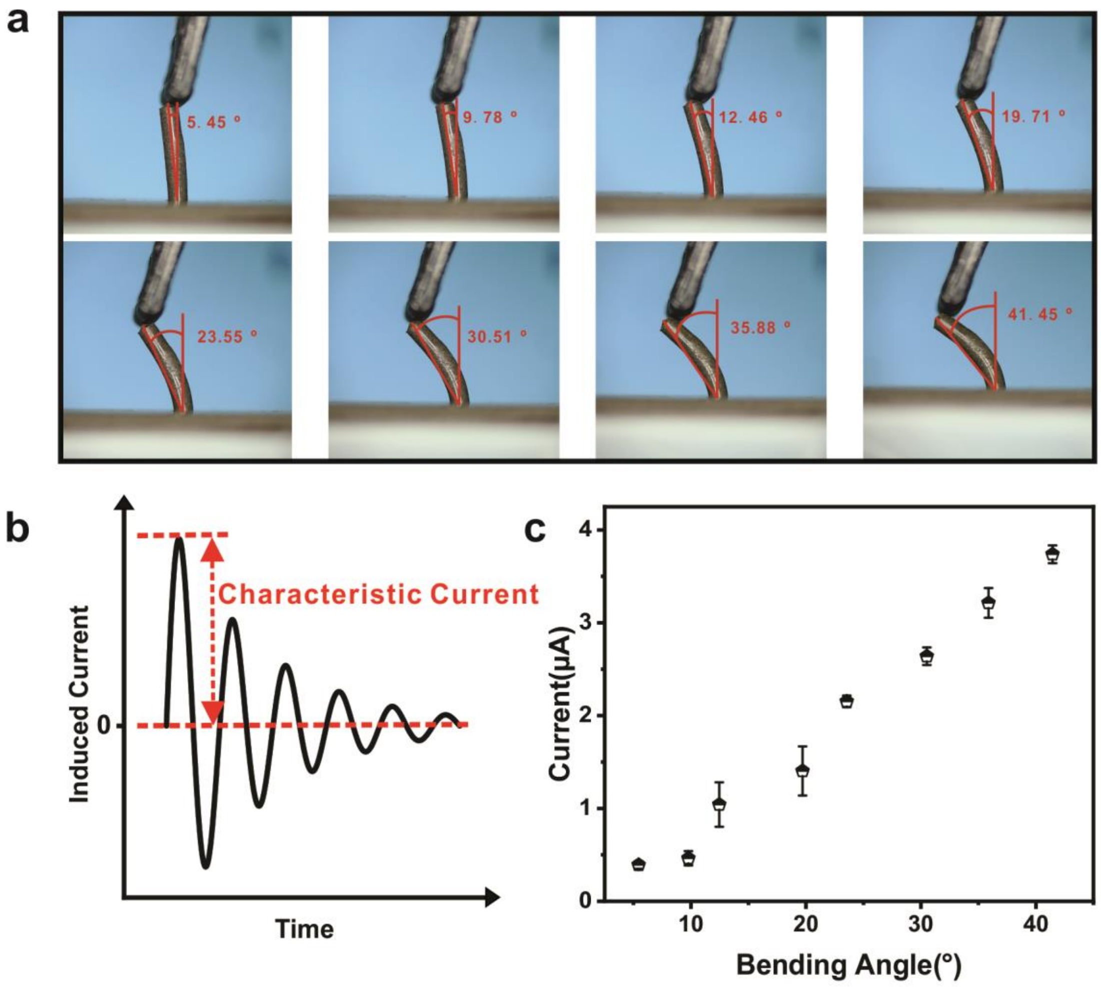

Supplementary Fig. 7. Influence of the bending angles of MMP on induced current during the oscillation process.

a, Bending angles of MMP driven by the blade. The relative vertical position of the platform was tuned to change the contact between the blade and the MMP for different bending angles. **b**, Schematic diagram of the characteristic current during the oscillation process for comparison. **c**, Corresponding characteristic current magnitude produced at different bending angles.

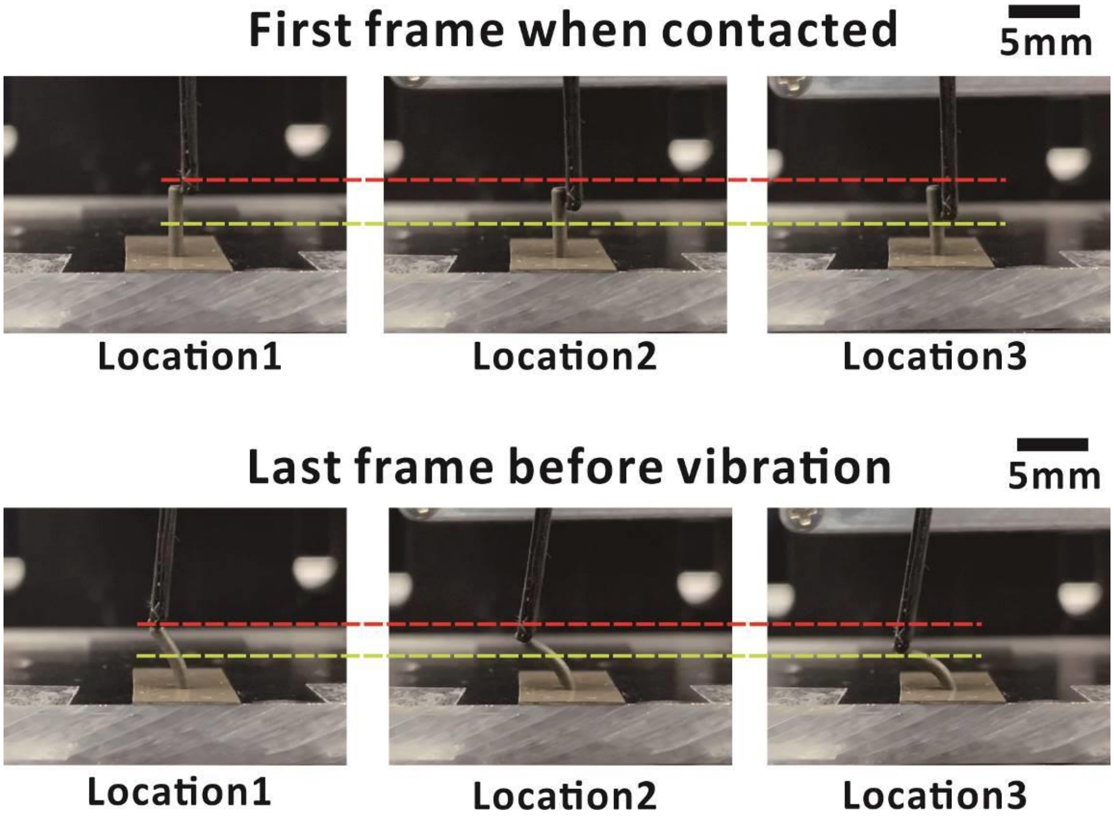

Supplementary Fig. 8. Optical images of three vibrating locations.

Vertical position of H5.0P0.5E0.5 could be flexibly adjusted, thus the blade can vibrate the MMP at different locations. If the blade impacts the micropillar with a relatively lower position, a larger deformation degree will be obtained once the blade leaves the micropillar.

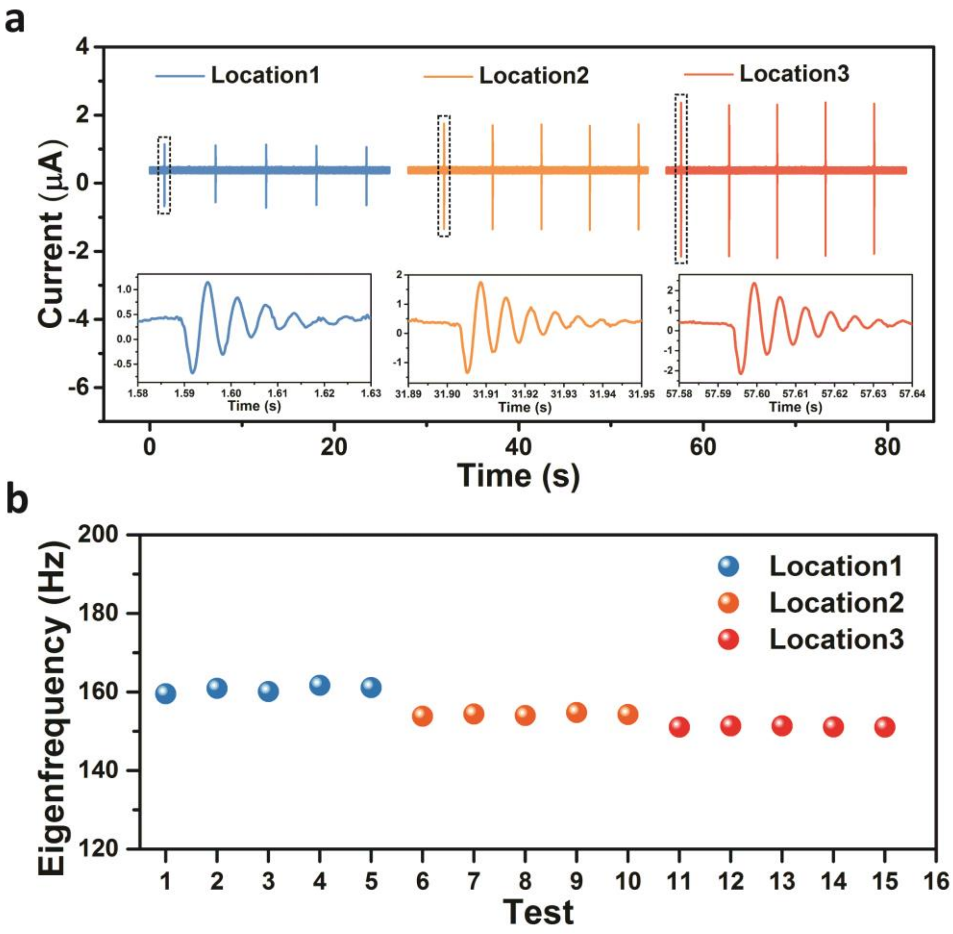

Supplementary Fig. 9. Oscillating signals corresponding to three impact locations.

a, Induced current within the coil when H5.0P0.5E0.5 was vibrated at three different locations. **b**, Comparison of eigenfrequencies of oscillating signals in (a).

Herein, we applied the blade to impact the micropillar at three different locations (**Fig. 2c** in the main context). The different locations of impact can affect the signal amplitude, which is attributed by the deformation degree. However, the eigenfrequency remains almost unaffected because of the inherent property (height, radius, modulus, and density, etc.) of the MMP is the same for all measurements.

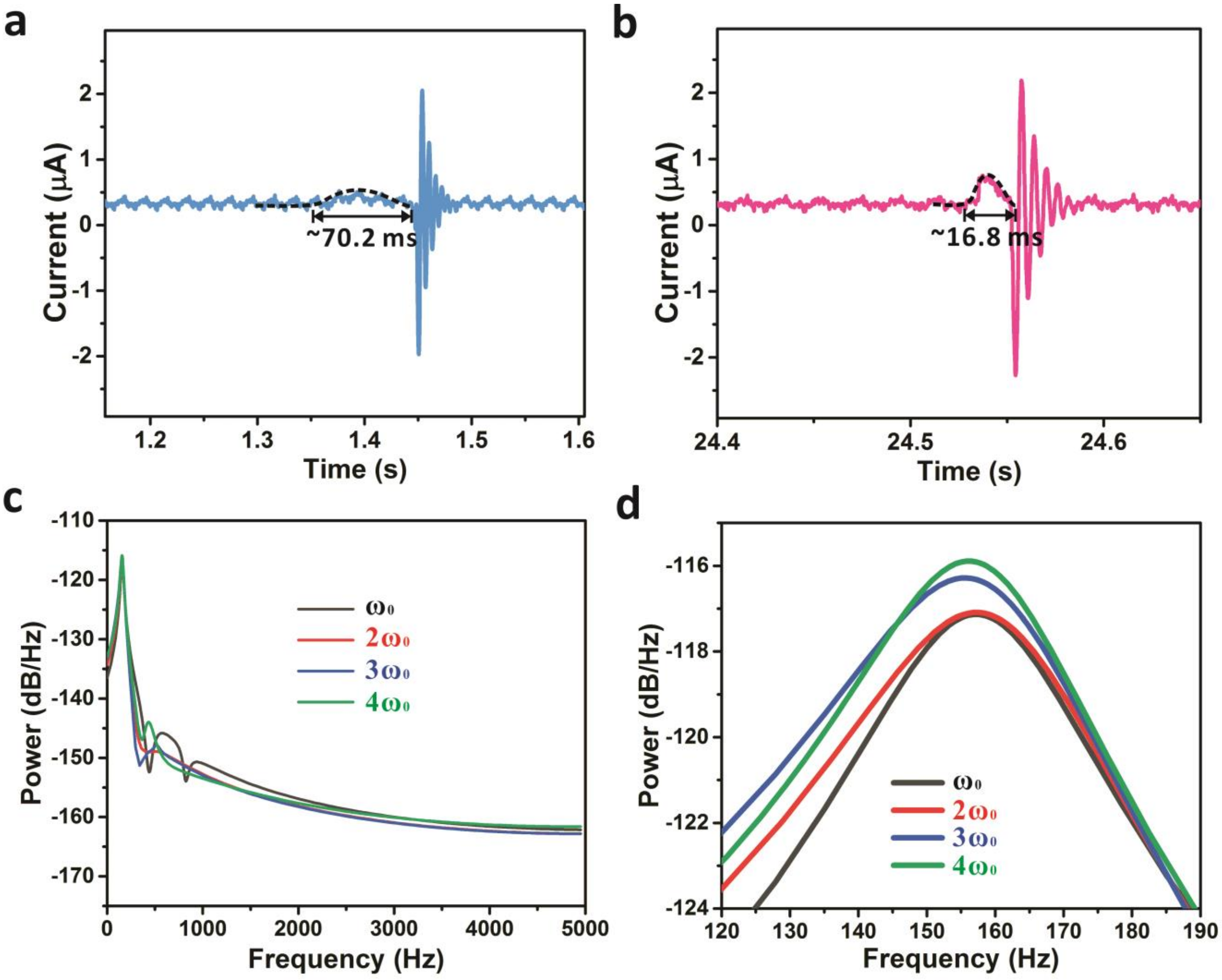

Supplementary Fig. 10. Oscillation of MMP with different vibrating speeds.

a, Induced current with vibrating speed of ω_0. **b**, Induced current with vibrating speed of $4\omega_0$. **c**, Prony energy spectrum of oscillation at different vibrating speeds of ω_0, $2\omega_0$, $3\omega_0$, and $4\omega_0$. **d**, Zoom-in Prony energy spectrum.

Supplementary Fig. 10a and **Supplementary Fig. 10b** show the induced current within the coil when the blade vibrates H5.0P0.5E0.5 at ω_0 and $4\omega_0$, respectively. The peak width which is corresponding to the deformation is about 70.2 ms when the motor speed is ω_0, while the peak width corresponding to the deformation is about 16.8 ms when the motor speed is $4\omega_0$, which is consistent with the change of the sweeping velocities. However, the waveform of intrinsic oscillation are basically the same, which indicates the oscillation is the inherent property of the studied micropillar. The zoom-in Prony energy spectrum also shows the eigenfrequencies locates almost at the same location and the influence from the sweeping speed can be ignored.

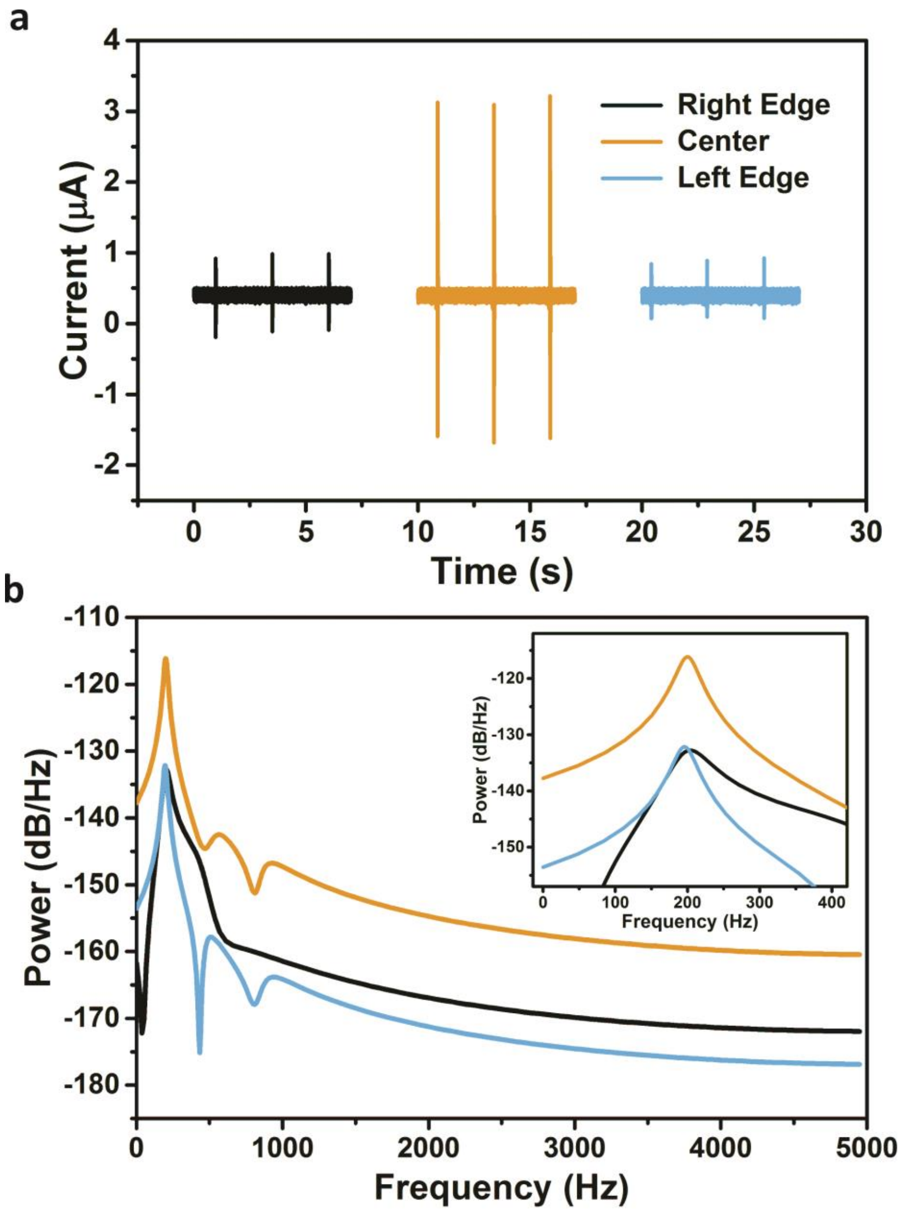

Supplementary Fig. 11. Oscillation signal when H5.0P0.5E0.5 was deposited on different locations above the coil.

a, Signals resulted from consecutive vibration when MMP was deposited on three different locations above the coil. **b**, Prony energy spectrum of different signals. The consistence of the peak frequency location indicates the eigenfrequency is determined by the inherent property of the MMP.

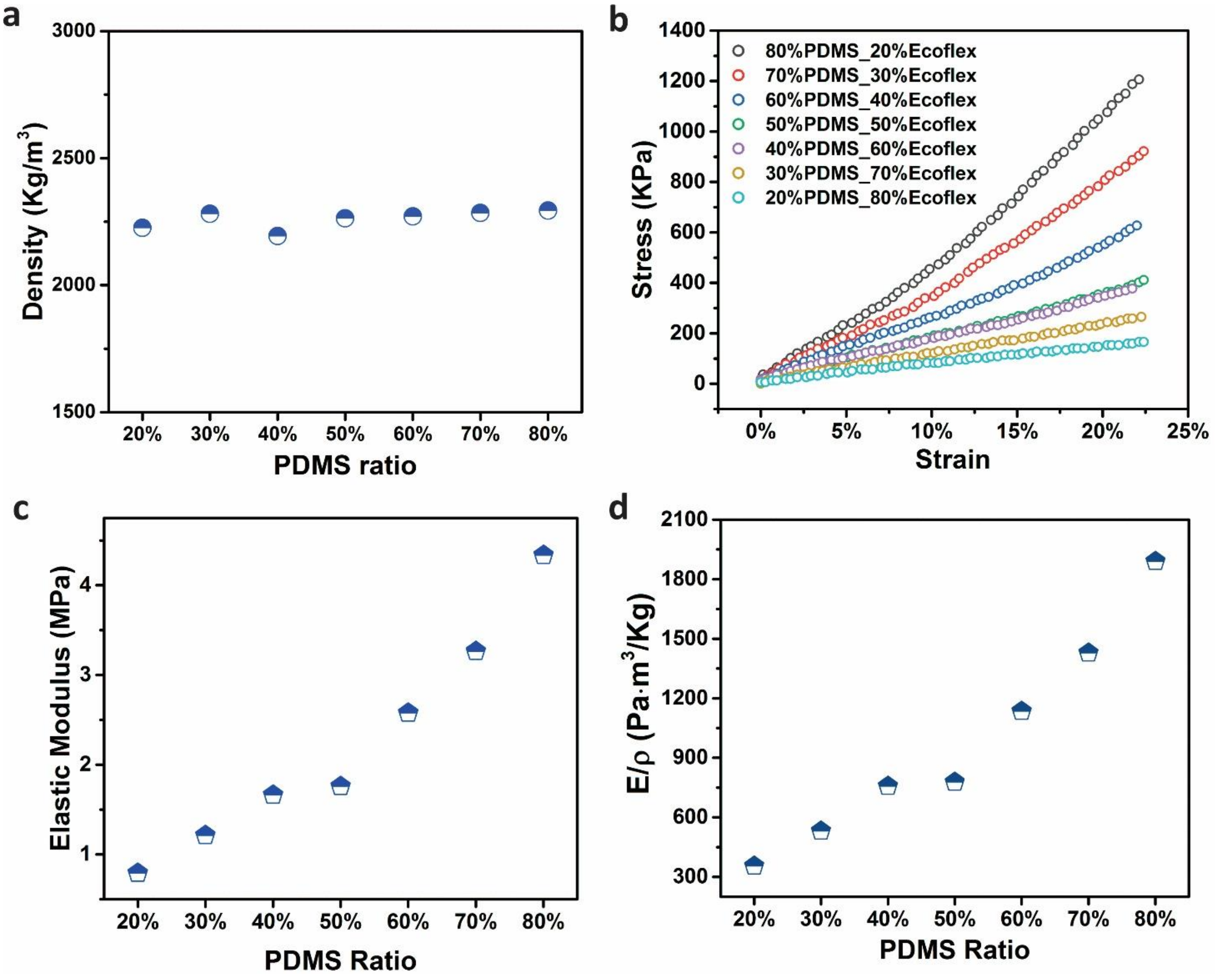

Supplementary Fig. 12. Parameters of materials with different components. The mass ratio of silicone polymer and NdFeB particles is fixed at 1:2, while the mass ratio of PDMS and Ecoflex is regulated.

a, Density of materials with silicone polymers based on different PDMS contents. **b**, Stain-stress curvature of different materials based on changing mass ratios of PDMS and Ecoflex. **c**, Elastic modulus of different materials with different ratios within the silicone polymers. **d**, Results of elastic modulus over density (E/ρ) based on different silicone polymers.

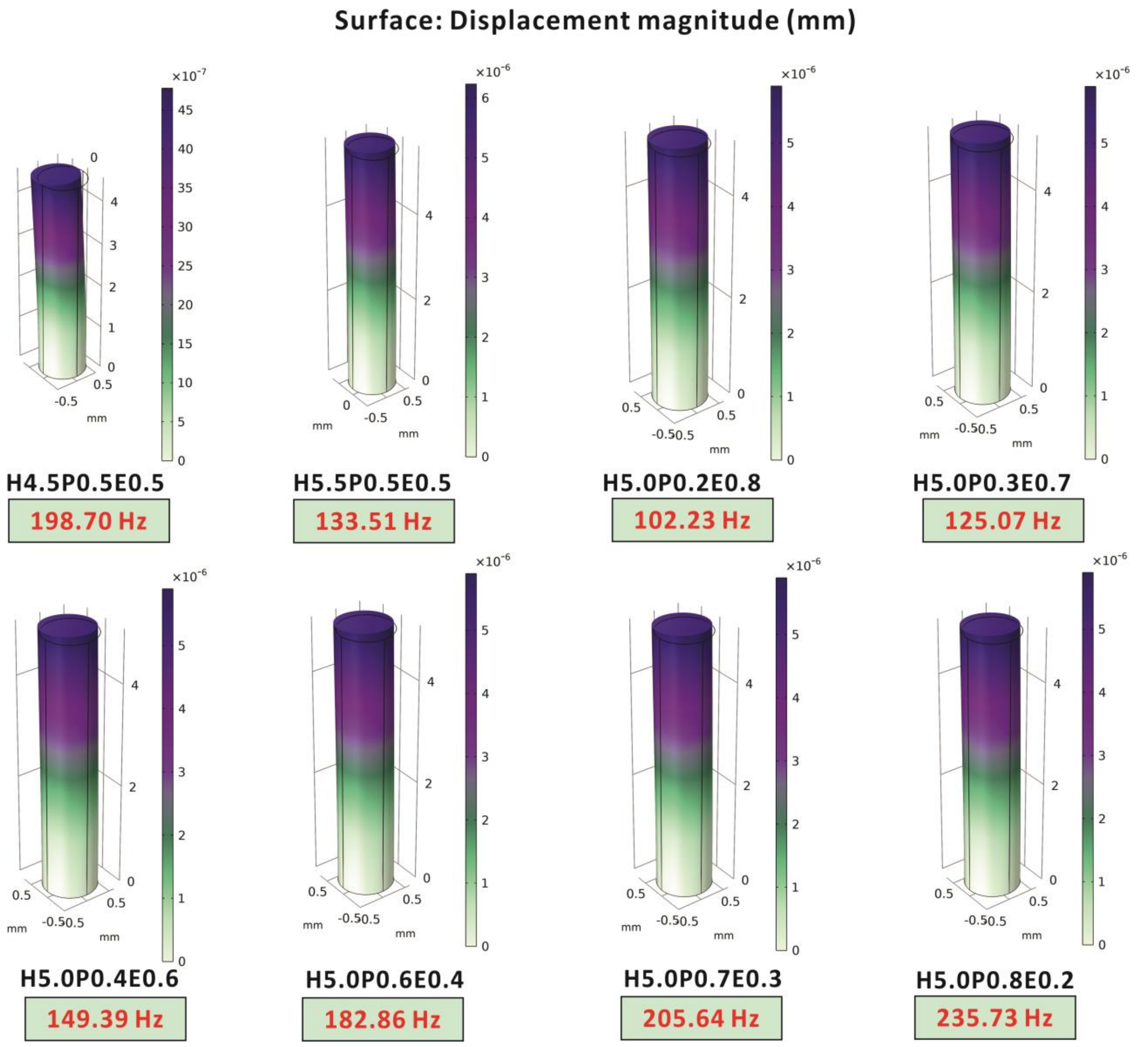

Supplementary Fig. 13. Simulated eigenfrequency of MMP with different parameters.

The obtained eigenfrequencies were indicated accordingly, which shows that the frequency values can be flexibly regulated via tuning the material property or the dimension of the micropillar.

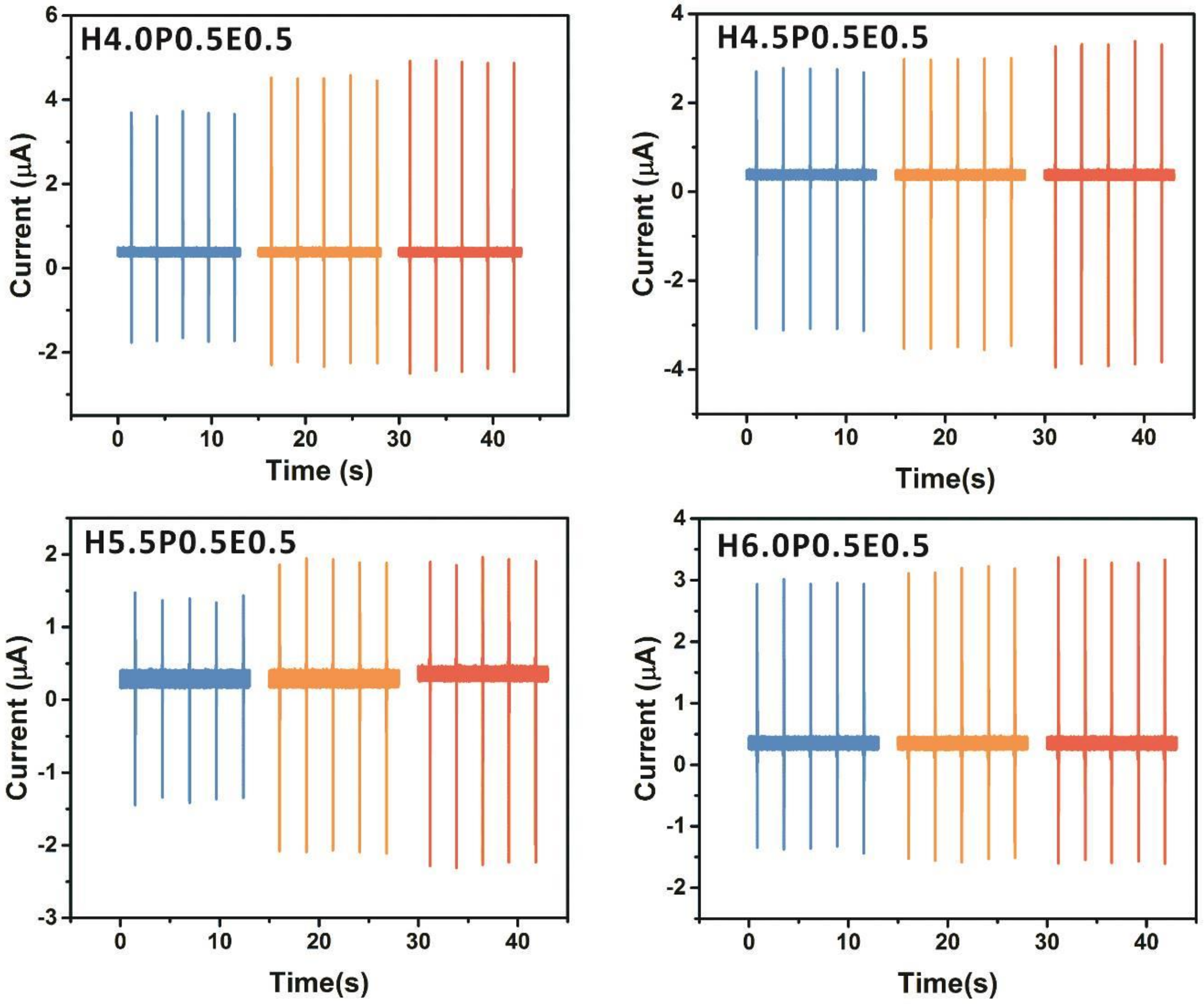

Supplementary Fig. 14. Vibration signals of MMPs with different values of *H*.

Three positions are selected for investigation for each type of the micropillar. The typical frequencies were then obtained by the average values from the collected data.

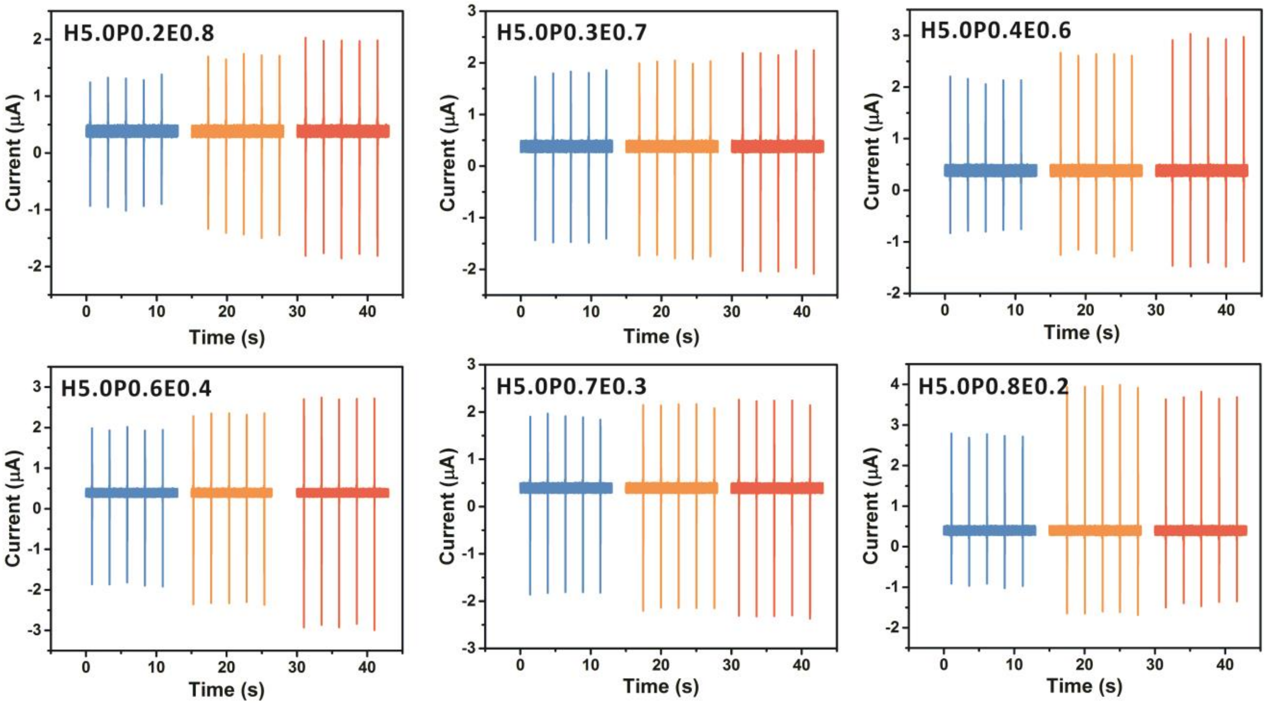

Supplementary Fig. 15. Vibration signals of MMPs with different material properties, *E/ρ*.

Three positions are selected for investigation for each micropillar. The typical frequencies were then obtained by the average values from the collected data.

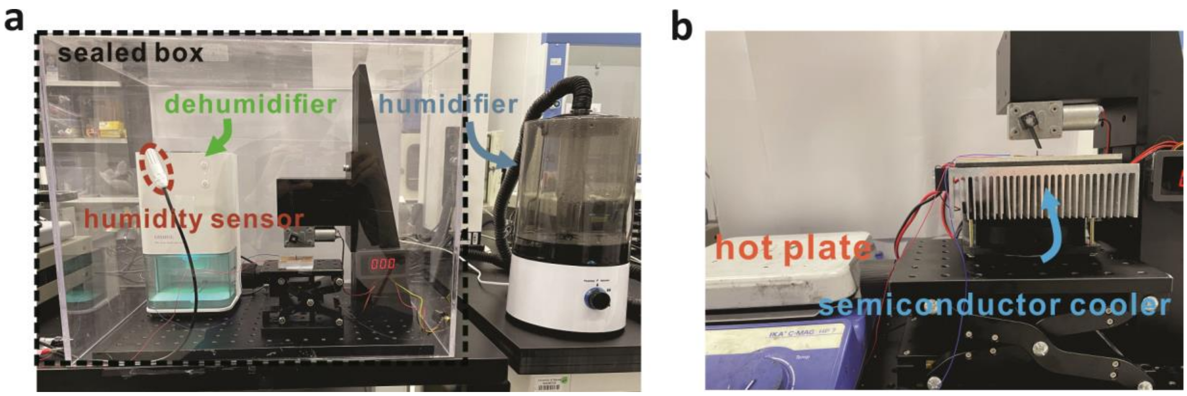

Supplementary Fig. 16. Experimental setups to measure the robustness of MMPs under different humidity and temperature conditions.

a, The characterization system with variable environmental humidity consists of a dehumidifier, humidity sensor, and a humidifier. The wearable interface was placed on the platform and the blade was used to deform the MMP. **b**, A hot-plate and a semiconductor cooler were applied to test the

temperature effect on the signal stability. Built-in temperature sensors were used to monitor the real-time temperature on the hot-plate and the semiconductor cooler.

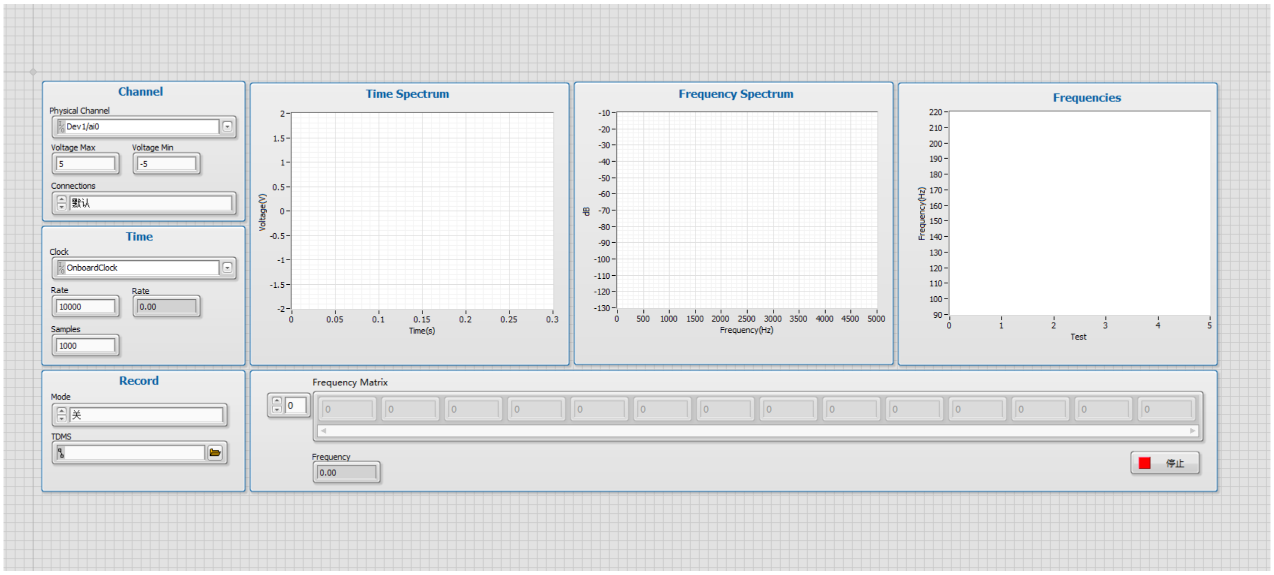

Supplementary Fig. 17. Software script interface.

The script is used to record the induced current within the coil in time domain and frequency domain, respectively. The interface can also display all the eigenfrequencies which is corresponding with all the oscillation process.

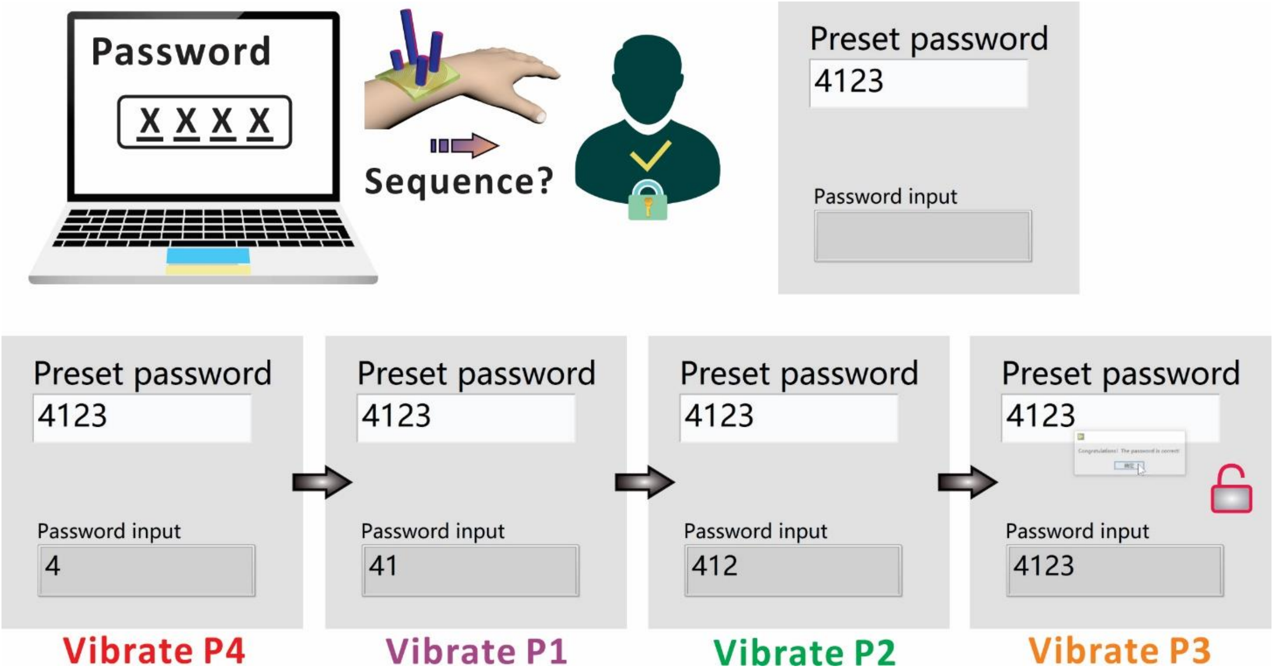

Supplementary Fig. 18. Password "4123" inputting and unlocking process.

A password "4123" was pre-defined in the software interface. When the human finger vibrated the micropillar in sequence of P4, P1, P2, and P3, the corresponding password "4123" can be generated. The matching of password allows the unlocking of the interface.

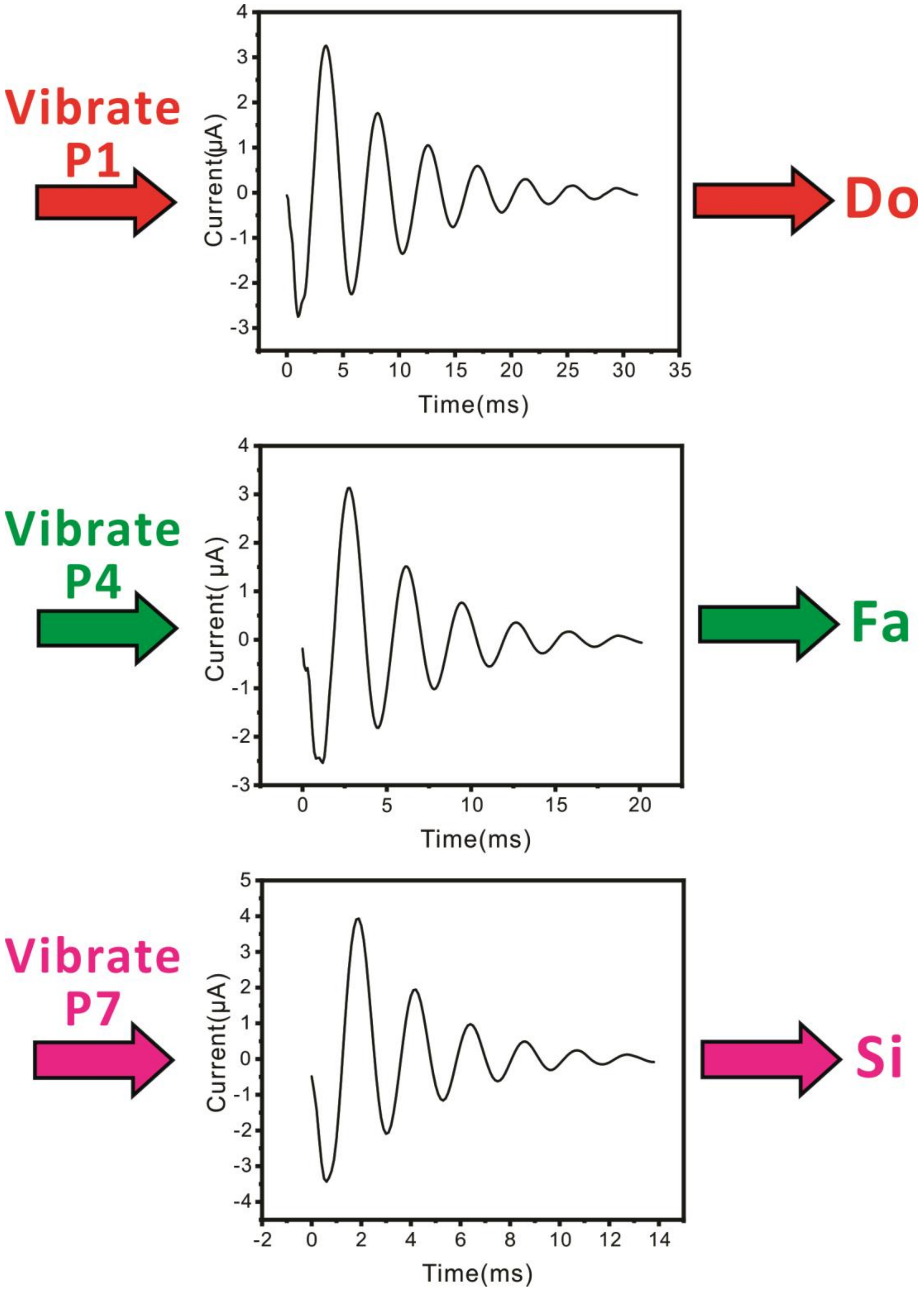

Supplementary Fig. 19. Production of electronic musical tones “Do”, “Fa” and “Si” based on the vibration of P1, P4, and P7.

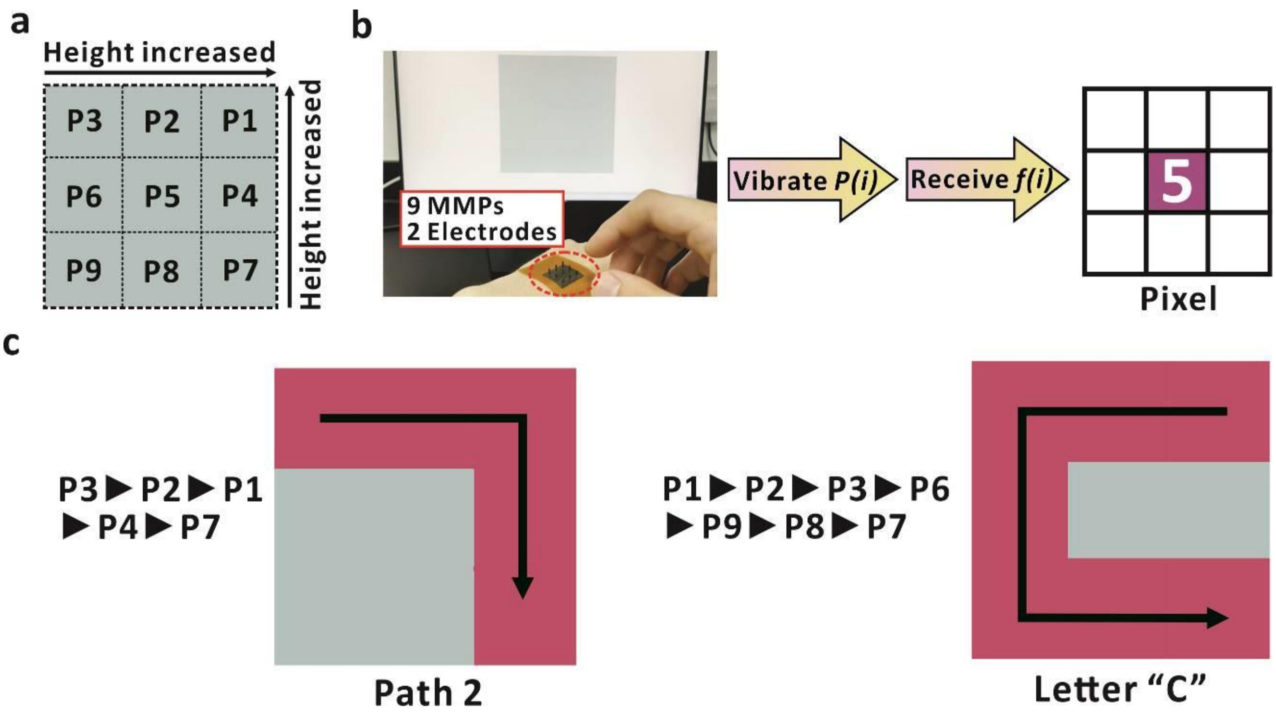

Supplementary Fig. 20. a, Distribution of the micropillar heights for the 3×3 arrays. **b**, Schematic diagram of the pixel addressing based on the MMP vibration and receiving of the related eigenfrequency. For example, if the received eigenfrequency is allocated to address the pixel 5, the corresponding pixel will be highlighted as depicted. **c,** Trajectory demonstrations to produce the path 2 and the letter "C" via vibrating the micropillars successively as indicated.

Supplementary Tables.

Table S1. Fitting parameters of the oscillating signal which are related with the data shown in **Fig. 2e**.

Component	Frequency	Amplitude	Phase constant	%
1	131.83 Hz	1.34e-6	4.32	16.95
2	161.15 Hz	1.36e-6	1.95	83.03
3	1055.31 Hz	4.92e-8	2.74	0.01

Table S2. Material properties based on the different mass ratios of PDMS and Ecoflex.

PDMS Mass Ratio	E (Pa)	P (Kg/m^3)	E/ρ ($Pa·m^3/Kg$)
80%	4334728	2294	1889.594
70%	3264692	2284.8	1428.874
60%	2577184	2270.7	1134.973
50%	1759693	2263	777.5929
40%	1662464	2193.4	757.9392
30%	1212600	2280.9	531.6324
20%	791201.4	2225.7	355.4843

Supplementary Videos.

Supplementary Video 1. Oscillating process of H4.0P0.5E0.5 and H6.0P0.5E0.5. A tweezer was manually controlled to deform the micropillar. The tweezer slowly approached the MMP, then bent the MMP to certain direction, and finally separated from the MMP. After that, MMP would like to vibrate around the equilibrium position with certain frequency. The video was filmed at 5000 fps and sped down to 0.006× for replay (i.e. 1 s in the video equals 6 ms in real time).

Supplementary Video 2. Consecutive vibration of four different micropillars and mapping demonstration. Four micropillars (H4.0P0.5E0.5, H4.5P0.5E0.5, H5.5P0.5E0.5, and H6.0P0.5E0.5) with different heights were attached on a conductive copper coil. When P1 was vibrated, signals induced within the coil with corresponding eigenfrequency were processed by a LabVIEW software script through a preamplifier and a DAQ (Data Acquisition). After signal processing, the number "1" would be illuminated. Similarly, when P2, P3, P4 were vibrated, the corresponding numbers would be illuminated.

Supplementary Video 3. Accuracy of the developed interface. Five volunteers were invited to continuously vibrate the micropillars H4.0P0.5E0.5 and H4.5P0.5E0.5 for 50 cycles, and the accuracy of the command recognition was tracked by the software simultaneously. The volunteers were briefly explained about the principle and the interface design, and they performed the test based on their daily operation habit.

Supplementary Video 4. Robotic control based on the developed interface. Eigenfrequencies of four MMPs were encoded with different operations of the robotic arm. When P1 was vibrated, electrical signals were transmitted via preamplifier and DAQ to the LabVIEW software script, which could process and transmit the signal to the robotic arm. After that, a "Move down" action could be realized by the robotic arm. Similarly, when P2, P3 and P4 were vibrated, the robotic arm would perform "Grasp", "Rotate right", and "Release", respectively. The combination of multiple commands enables the tomato to be grasped and transferred.

Supplementary Video 5. Password input and unlocking process via the MMP-based system. Eigenfrequencies of four MMPs were encoded with different number inputs. When P1 was vibrated, electrical signal with specific eigenfrequency would be transmitted to the software script via preamplifier and DAQ, and the number "1" would be inputted into the "Password input" panel. Similarly, when P2, P3 and P4 were vibrated, numbers "2", "3", and "4" would be inputted, respectively. When the numbers in "Password input" panel are the same with "Preset password" panel, it would be unlocked. Two different passwords, "4123" and "1432", were demonstrated.

Supplementary Video 6. Seven MMPs-based system for generation of musical tones. Seven micropillars were attached on the flexible coil in a row, and eigenfrequency of 7 micropillars was encoded with different key pressing based on a LabVIEW program. For example, when P1 (height of 4.0 mm) was vibrated, the key "Do" would be pressed and highlighted. The video records the

successive vibration from P1 to P7, and the corresponding keys “Do”, “Re”, “Mi”, “Fa”, “Sol”, “La”, and “Si” were pressed and highlighted in turn.

Supplementary Video 7. Nine MMPs-based system for trajectory monitoring and interactive writing. Nine micropillars with different heights were attached on the flexible coil. The MMPs were arranged in 3×3 array. Each MMP corresponds to a square area in the LabVIEW program, and the corresponding area would be highlighted when a particular MMP is vibrated. When the finger vibrates the pillar in different positions, the corresponding area is highlighted, so that the trajectory of the finger operation can be visualised on the screen. The video records two different paths and the letters of “C” and “U”, which are produced via vibrating the MMPs in a specific sequence.

Supplementary Note 1. Prony Method for Signal Analysis.

Prony method is a signal processing technique based on signal estimation, which extracts desired information from an equally spaced sampled signal and builds a series of damped complex exponentials to approximate the sampled signals by solving a set of linear equations.[1–3]

Prony method defines the parameters of a signal model $y(t)$ in (2), and they can be obtained by sampling the signal and obtaining the data samples $[y(1),\ y(2), \dots,\ y(n)\ ]$ using a sampling frequency f_s.

$$y(t) = \sum_{n=1}^{N} A_n e^{\sigma_n t} \cos(2\pi f_n t + \theta_n) \qquad (2)$$

The Prony model signal approximates the sampled data in (2) using the following linear combination of p complex exponentials:

$$y_M = \sum_{n=1}^{p} B_n \lambda_n^M \qquad (3)$$

$$B_n = \frac{A_n}{2} e^{j\theta_n}$$

$$\lambda_n = \frac{A_n}{2} e^{(\sigma_n + j2\pi f_{n)T}}$$

The signal $y(t)$ in (2) has four elements: magnitude A_n, damping factor σ_n, frequency, and the phase angle θ_n. Each exponential term is a unique signal mode of the original signal $y(t)$. Consequently, using the Euler theorem and total time $t = MT$, where M is the length of the signal and T is the time between samples, Equation (2) can be rewritten as (3).

Note that the Prony method to be implemented in online test diagnostics (OTD) for rotor cage induction motor (RCIM)[3], the following steps should be considered, which could also be used here for signal analysing:

(1) Know the sampling frequency (f_s), sampling time (T_s), length of the current signal under analysis (L) with a minimum of 25 cycles of data and the order (p) of the linear prediction model (LMP), where an initial value of p for a data window of current signal measurement for analysis must be selected.

(2) A Toeplitz matrix "ϒ" with the data of the current signal "$y(t)$" must be defined as (4).

$$\Upsilon = \begin{bmatrix} y[p] & y[p-1] & \dots & y[1] \\ y[p+1] & y[p] & \dots & y[2] \\ \vdots & \vdots & \ddots & \vdots \\ y[2p-1] & y[2p-2] & \dots & y[p] \end{bmatrix} \qquad (4)$$

(3) A vector "a" (coefficients of characteristic Equation (3)) using (4) is calculated in (5).

$$\begin{bmatrix} a[1] \\ a[2] \\ \vdots \\ a[p] \end{bmatrix} = \begin{bmatrix} y[p] & y[p-1] & \dots & y[1] \\ y[p+1] & y[p] & \dots & y[2] \\ \vdots & \vdots & \ddots & \vdots \\ y[2p-1] & y[2p-2] & \dots & y[p] \end{bmatrix} \cdot \left(-\begin{bmatrix} y[p+1] \\ y[p+1 \\ \vdots \\ y[2p] \end{bmatrix} \right) \tag{5}$$

(4) Calculate the roots from vector "a" and the resulting roots vector "z" will be used in (6) and (7) to calculate the damping

$$\sigma = \frac{ln|z|}{T_s} \tag{6}$$

and frequency

$$f = \frac{1}{2\pi T_s} \tan^{-1}\left(\frac{Im(z)}{Re(z)}\right) \tag{7}$$

Supplementary Note 2. Analysis of cylindrical cantilever vibration.

In this study, the MMP was fixed at one end and the other end was set at free. We thus employ the cylindrical cantilever beam to build up the corresponding model. Free vibration of an equal section Euler-Bernoulli beam[4] is governed by the formula:

$$EJ\frac{\partial^4 y}{\partial x^4} + \rho A\frac{\partial^2 y}{\partial x^2} = 0 \tag{8}$$

where E is elastic modulus, J is the cross sectional moment of inertia, ρ is the material density, and A is the cross-sectional area. The main vibration pattern of the beam can be assumed as:

$$y(x,t) = Y(x)b\,sin(wt + \varphi) \tag{9}$$

where $Y(x)$ is main vibration pattern function of the beam, substituting Eq. (9) into Eq. (8), we would get:

$$Y^{(4)} - \beta^4 Y = 0 \tag{10}$$

where

$$\beta^4 = \frac{\omega^2}{a^2}, a^2 = \frac{EJ}{\rho A}$$

The general solution of Eq. (10) is

$$Y(x) = C_1\, cos(\beta x) + C_2\, sin(\beta x) + C_3\, ch(\beta x) + C_4\, sh(\beta x) \tag{11}$$

The boundary conditions of the cantilever beam are:

$$Y(0) = Y'(0) = 0$$

$$Y''(l) = Y'''(l) = 0$$

Consequently, the eigenfrequency of the cantilever beam is:

$$\omega_n = \frac{K^2}{l^2}\sqrt{\frac{EJ}{\rho A}} \tag{12}$$

where K values are corresponding to different orders of the eigenfrequencies of the cantilever beam, and the first order K is 1.875. The cross-sectional moment of inertia J of a cylinder is $J = \frac{\pi R^4}{4}$, and the cross-section area $A = \pi R^2$. In our case (cylindrical cantilever beam), l is the height of the micropillar, h.

The 1st eigenfrequency of the cylindrical cantilever beam is finalized as:

$$f = \frac{1.875^2}{4\pi h^2}\sqrt{\frac{ER^2}{\rho}} \tag{13}$$

References.

1. Lobos, T., Reziner, J. & Schegner, P. Parameter estimation of distorted signals using prony method. in *2003 IEEE Bologna Power Tech Conference Proceedings,* vol. 4 692–696 (IEEE, 2004).

2. Kay, S. M. & Marple, S. L. Spectrum Analysis-A Modern Perspective. *Proc. IEEE* **69**, 1380–1419 (1981).

3. Guajardo, L. A. T. *et al.* Prony Method Estimation for Motor Current Signal Analysis Diagnostics in Rotor Cage Induction Motors. *Energies* **15**, (2022).

4. Kurmendra & Kumar, R. Design analysis, modeling and simulation of novel rectangular cantilever beam for MEMS sensors and energy harvesting applications. *Int. J. Inf. Technol.* **9**, 295–302 (2017).
